# Molecular Choreography and Structure of Ca^2+^ Release-Activated Ca^2+^ (CRAC) and K_Ca2+_ Channels and Their Relevance in Disease with Special Focus on Cancer

**DOI:** 10.3390/membranes10120425

**Published:** 2020-12-15

**Authors:** Adéla Tiffner, Isabella Derler

**Affiliations:** Institute of Biophysics, JKU Life Science Center, Johannes Kepler University Linz, A-4020 Linz, Austria; adela.tiffner@jku.at

**Keywords:** STIM1, Orai1, CRAC channels, store-operated channel activation (SOCE), Ca^2+^-activated K^+^ channels (K_Ca2+_), SK3, SK3-Orai1 interplay

## Abstract

Ca^2+^ ions play a variety of roles in the human body as well as within a single cell. Cellular Ca^2+^ signal transduction processes are governed by Ca^2+^ sensing and Ca^2+^ transporting proteins. In this review, we discuss the Ca^2+^ and the Ca^2+^-sensing ion channels with particular focus on the structure-function relationship of the Ca^2+^ release-activated Ca^2+^ (CRAC) ion channel, the Ca^2+^-activated K^+^ (K_Ca2+_) ion channels, and their modulation via other cellular components. Moreover, we highlight their roles in healthy signaling processes as well as in disease with a special focus on cancer. As K_Ca2+_ channels are activated via elevations of intracellular Ca^2+^ levels, we summarize the current knowledge on the action mechanisms of the interplay of CRAC and K_Ca2+_ ion channels and their role in cancer cell development.

## 1. Introduction

Ion channels form hydrophilic pores in the cell membrane and allow selective permeation of ions of appropriate size and charge across the membrane down their electrochemical gradient. This process is accomplished via diverse intrinsic ion channel gating mechanisms in response to a specific stimulus like a change in the membrane potential or binding of a neurotransmitter. Hence, electrical or chemical stimulation of ion channels leads to their transient opening. The plethora of cell functions is fulfilled by the action of single ion channels and their interplay with a variety of other cellular components. They are often clustered in signal complexes in the membrane, known as signalplexes, to enable efficient signal transduction. Moreover, the interplay of different kinds of ion channels is crucial for proper cell function, a diversity of essential intracellular signaling cascades and downstream processes such as proliferation, differentiation, motility, and apoptosis [1,2,3,4,5,6,7,8,9,10,11].

Dysfunctions of ion channels can lead to a diversity of diseases, for instance of the nervous system or the muscle, and thus, have been called channelopathies [1,2,4,5,12,13]. In addition to defects in ion channel function, altered expression levels or interplay of ion channels strongly correlates with pathological characteristics and can provoke various extents of diverse channelopathies [14,15,16,17,18]. This is the case for certain Ca^2+^-regulated ion channels [8,19], which are the focus of this review.

In the following, we initially highlight the role of Ca^2+^ ions in the cell.

## 2. Ca^2+^ Signaling

Calcium (Ca^2+^) ions are universal and remarkably versatile secondary messengers that control a plethora of cellular functions and processes including gene transcription, proliferation, and cell migration. They govern major steps throughout the cell’s life cycle defining whether the cell should live, divide, move, or die [20,21,22]. They trigger fundamental biological processes such as the release of neurotransmitters of neurons or inflammatory mediators of immune cells [20,21,23,24,25,26,27]. At resting conditions, the cytosolic Ca^2+^ concentration ([Ca^2+^]_i_) is kept at very low basal levels around 10^−7^ M [28]. This ensures a vast dynamic range of elevated Ca^2+^ concentrations necessary and triggers a diversity of cellular Ca^2+^ signals [29].

There are two major sources for Ca^2+^ in the cell: on the one hand intracellular compartments, such as the endoplasmic reticulum (ER) or the mitochondria, that can deplete stored Ca^2+^ into the cytosol and on the other hand, the extracellular side of the cell from where Ca^2+^ ions can enter into the cell in response to a specific signal. Alterations in the cellular Ca^2+^ levels are tightly controlled by a set of Ca^2+^ signaling proteins including Ca^2+^ transporters, Ca^2+^ regulated ion channels, Ca^2+^-binding proteins, Ca^2+^-storage proteins, and Ca^2+^-dependent effectors. They act with highly spatiotemporal dynamics to govern the respective cellular processes in a cell-type-specific manner. Ca^2+^ signals often occur in the form of global calcium oscillations, waves or short-lived local Ca^2+^ sparks, spikes or flickers [20,21,30,31].

Defects in the cellular Ca^2+^ homeostasis due to dysfunction or changes in the occurrence of one of these molecular key players in Ca^2+^ signal transduction can be responsible for severe immune deficiencies, neuronal diseases or heart problems. Moreover, altered Ca^2+^ signaling can lead to various cancers by regulating one or more of the known cancer hallmarks [19,32,33] such as sustained proliferation signals, apoptosis resistance, evading growth suppressors, induction of angiogenesis, replicative immortality, tissue invasion, and metastasis cancer hallmarks [6,7,8,32,34,35,36,37,38,39,40,41,42,43,44].

A detailed picture of the working mechanisms of Ca^2+^ signaling molecules is essential to develop new therapeutic and preventive methods against diseases related to the respective Ca^2+^ regulated proteins [1,2,3,5,8].

In the following sections, we highlight the functionally relevant key structures and activation mechanisms of Ca^2+^-sensitive ion channels, specifically, the calcium release-activated calcium (CRAC) channel and the Ca^2+^-activated K^+^ ion channels (K_Ca2+_), individually as well as when they interplay or act in synergy. Detailed knowledge of the precise working mechanisms of individual ion channels provides novel directions to enhance our understanding of their co-regulation and to characterize so far elusive molecular determinants for their interplay. Thus, a basis for the development of more specific therapeutic strategies against diverse diseases such as cancer can be established.

### Calcium Ion Channels

Ca^2+^ channels conduct Ca^2+^ ions down the electrochemical gradient without the use of energy. Generally, Ca^2+^ permeates from areas with higher Ca^2+^ concentrations, such as the extracellular space or the intracellular stores (ER), to those with lower Ca^2+^ concentration like the cytoplasm. Ca^2+^ ion channels are either located in the plasma membrane or the membrane of intracellular organelles, such as the ER, mitochondria, or lysosomes [8,45]. Most prominent Ca^2+^ ion channels represent voltage-gated channels (VGCC) [46], ligand-gated channels (LGC) [47], store-operated channels (SOC) [48], transient receptor potential channels (TRP) [49], and mechanically gated channels [50]. There is accumulating evidence that some members of those ion channel families are located in intracellular organelles [51]. Moreover, well-known Ca^2+^ ion channels in the intracellular stores include for instance the inositol trisphosphate receptor (IP_3_R) or the mitochondrial Ca^2+^ uniporter (MCU) [45,51,52,53,54]. This large variety of Ca^2+^ ion channels governs via highly specific Ca^2+^ signals a multitude of essential events in a cell’s life including gene expression, secretion, proliferation, differentiation, or migration. Furthermore, they control a plethora of healthy processes such as muscle contraction, neuronal function, and immune cell function [20,21,55]. Thus, it is not surprising, that defects in those signals can cause neuronal diseases, immune deficiencies [1,2,3,4,5,12,13,56], and cancer invasion [8,37,40,41,57,58,59,60,61,62,63,64,65].

Among this variety of Ca^2+^ channels, the focus in this review is laid on the SOC family, specifically the calcium release-activated calcium (CRAC) channel.

## 3. CRAC Channels

**Figure 1:**
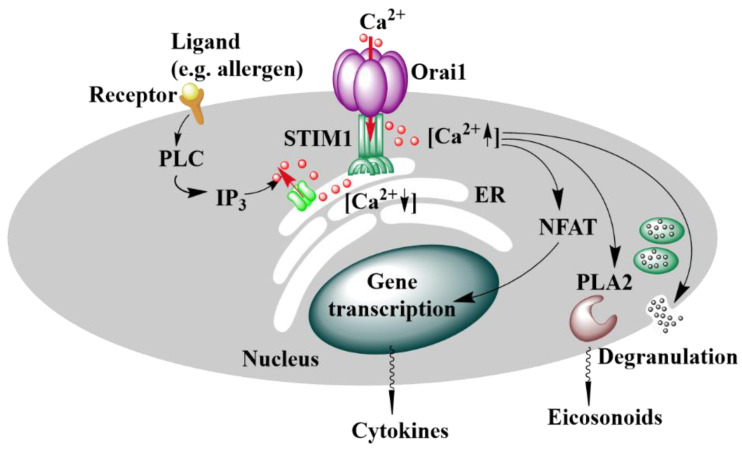
The CRAC channel activation pathway and downstream signaling processes. The scheme represents the activation pathways in the mast cell subsequent to the binding of a ligand to a receptor at the extracellular side. Activation of phospholipase C and generation of IP_3_ leads to store-depletion, activation of STIM1 and subsequent STIM1/Orai1 coupling. Ca^2+^ influx that occurs upon Orai1 opening mediates downstream signaling cascades that can lead to gene transcription or release of inflammatory mediators such as histamines, eicosanoids, and cytokines.

In non-excitable cells, store-operated (SOC) channels manifest a primary route for Ca^2+^ entry (SOCE) into the cell. Among SOC channels, Ca^2+^ release-activated Ca^2+^ (CRAC) channels are most prominent [48,66]. They are constituted by the ER-located Ca^2+^-sensing stromal interaction molecule STIM1 [57,67] and the Orai Ca^2+^ ion channel in the plasma membrane [68,69], an oligomeric complex of Orai subunits [70]. The family of STIM proteins includes STIM1 and STIM2, while the Orai family contains Orai1, Orai2, and Orai3 [68,71]. Briefly, CRAC channel activation is initiated upon Ca^2+^ depletion from the internal stores, which can occur via IP_3_R [48,72] (Figure 1). The reduction in the Ca^2+^ concentration is detected via STIM1 [73,74,75,76], which, subsequently undergoes a conformational change, oligomerizes, forms junctions toward the plasma membrane and activates the highly Ca^2+^-selective ion channel, Orai1 [73,77,78,79,80].

Orai1 and STIM1 trigger together primarily immune cell function [77], however, they are furthermore involved in the regulation of a diversity of other healthy processes in the human body (Figure 1) [55,81,82]. Moreover, STIM1 and Orai1 are upregulated in cancer cells and to trigger their development and growth [40,41,58,59,60,61,62,63].

### 3.1. STIM Proteins

In addition to the two homologs STIM1 and STIM2, additional splice variants STIM1L and STIM2.1, STIM2.3 are currently known [83,84,85]. Structurally, STIM proteins represent single-pass transmembrane proteins with the N-terminus embedded in the ER lumen. There, they detect fluctuations of Ca^2+^ levels and respond to those changes via conformational rearrangements to trigger Orai1 activation, mediating SOCE. The STIM1 protein is sensitive to more drastic changes in luminal Ca^2+^ levels with the dissociation constant, K_d_ = 200 μM, compared to STIM2, which detects smaller changes of Ca^2+^ concentrations (K_d_ = 500 μM) [86]. 

STIM1 as well as STIM2 plays an indispensable role in the immune system and are expressed in diverse primary lymphocytes such as T_h_, T_C_, and B-cells [56,87]. Nevertheless, they have been detected to be widely spread in a variety of other tissues [82,88,89,90,91]. High STIM1 levels have been reported for the heart, skeletal muscle, and the central nervous system [1,92,93,94]. Meanwhile, there is clear evidence that STIM1 proteins occur in a variety of cancer types [92], such a breast, cervical, glioblastoma, and colorectal cancers [92,95].

#### STIM1 Structure

**Figure 2:**
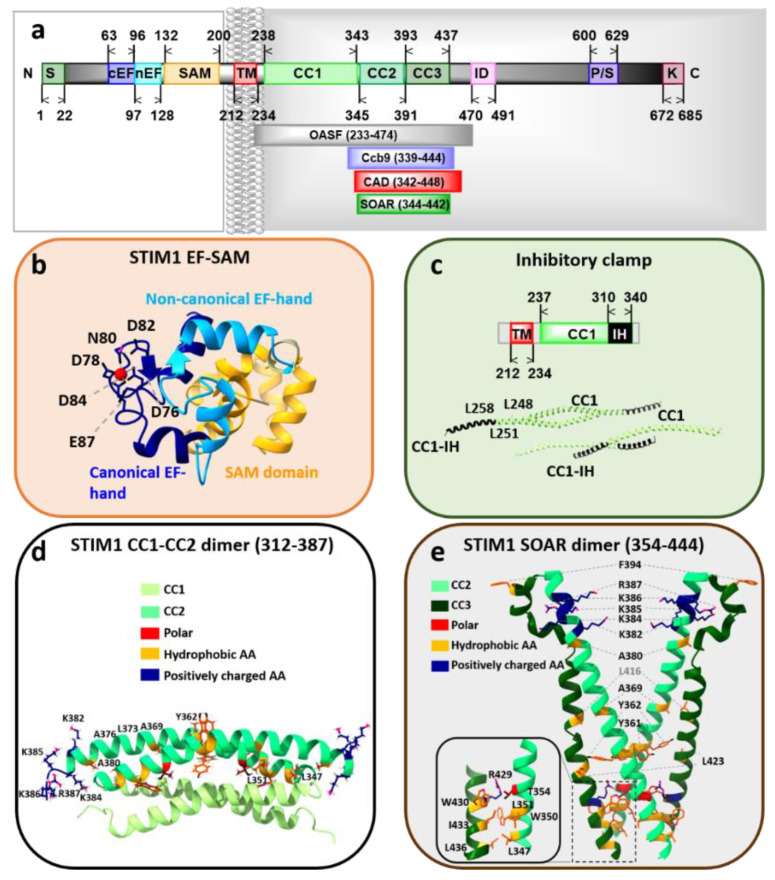
The structural features of Stromal interaction molecule 1 (STIM1). (**a**) Scheme displays the full-length human STIM1 with highlighted regions critical for the regulation of the STIM1/Orai1 signaling cascade. Important fragments, such as OASF, Ccb9, CAD, and SOAR are further shown as insets. (**b**) The high-resolution EF-SAM domain structure of human STIM1 loaded with a Ca^2+^ ion (red sphere) is displayed. The residues with proposed Ca^2+^-binding ability are highlighted. (**c**) The crystal structure of STIM1 CC1-inhibitory helix domain with the critical residues maintaining the STIM1 quiescent state are presented. (**d**) The NMR structure of a STIM1 CC1α3-CC2 dimer with the two monomers coupled in an antiparallel manner. Each monomer is bent with a sharp kink between the two coiled-coil domains. (**e**) The crystallographic structure of the STIM1 SOAR dimer, forming a V-shape. A single monomer comprises CC2 and CC3 domains and resembles the capital letter, “R.” Residues that represent potential interaction sites within the dimer and those mediating coupling to Orai1 are highlighted. Left inset: Magnified view of amino acids involved in dimer interactions between the N-terminal portion of the first SOAR monomer and the C-terminal portion of the second SOAR monomer.

The STIM1 protein is composed of 685 aa containing crucial regions specialized for Ca^2+^ sensing, the establishment of its quiescent or active state and Orai1 coupling (Figure 2a). The STIM1 luminal part consists of a signal peptide (aa: 1–22) at the beginning of the N-terminus which ensures its translocation to the ER [96,97]. The EF hand region (aa: 63–128) senses Ca^2+^ fluctuations in the sub-millimolar range and includes two EF hand motifs, a canonical (c, aa: 63–96) and a non-canonical (n, aa: 97–128) one [98]. The cEF hand coordinates the Ca^2+^ binding, whereas the nEF hand stabilizes the structure of the cEF hand. Negatively charged aspartates and glutamates within the cEF hand have been suggested to be involved in Ca^2+^ binding [75,98] (Figure 2b). The EF hand is connected to a sterile α-motif (SAM, aa: 132–200) via a short helical structure. The SAM domain forms a five α-helix bundle structure essential for store depletion-induced puncta formation. Under resting conditions, hydrophobic interactions between the EF hand region and the SAM domain establish the EF-SAM complex [99,100] (Figure 2b).

The N-terminus is followed by the single TM domain (aa: 212–234) of STIM1. It contains three glycine residues that provide structural flexibility to allow conformational changes upon STIM1 activation [101] (Figure 2a).

The STIM1 C-terminus (aa: 238–685) is indispensable for the coupling to the Orai1 channel. The C-terminus consists of three coiled-coil domains (CC1 aa: 238–337, CC2 aa: 345–391, and CC3 aa 393–437), the inhibitory (aa: 470–491) [102] or CRAC modulatory domain (aa: 474–485) [103], the Ser/Pro-rich region (aa: 600–629) [74] and the lysine-rich region (aa: 672–685) [96,104,105]. Diverse minimal fragments such as the Orai1-activating small fragment (OASF, aa: 233–474) [106], the CRAC-activating domain (CAD, aa: 342–448) [107], the coiled-coil domain containing region b9 (Ccb9, aa: 339–444) [108], and the STIM Orai-activating region (SOAR, aa: 344–442) [80] have revealed that CC2 and an extended portion of CC3 are sufficient for Orai activation (Figure 2a).

Among the structural resolutions of STIM1, the structure of CC1, the coiled-region subsequent to the TM, is available. It has been reported to contain an inhibitory helix potentially involved in the maintenance of the STIM1 quiescent state [109] (Figure 2c). Moreover, the SOAR-like fragments (aa: 354–444 [110] and, aa: 312–387 [111]) have been structurally resolved. Both C-terminal fragments appear in a dimeric assembly, however, their overall conformation is distinct. In detail, two monomers of the SOAR-like fragment aa: 354–444 resembling the capital letter **“**R**”** form the V-shaped dimer [110]. The monomers of the aa: 312–387 structure show one kink between CC1 and CC2 and cross each other in an antiparallel manner [111] (Figure 2d,e).

The monomers of the aa: 354–444 fragment consist of four α-helices, α1 (α1, (aa: 345–391), α2, (aa: 393–398), α3, (aa: 400–403), and α4, (aa: 408–437)). The first helix, α1 functions as the STIM-Orai association pocket (SOAP) [111]. α2 and α3 are involved in the coupling to and gating of Orai1 [1,112,113]. Under resting conditions, α4 is proposed to keep STIM1 in the quiescent state via an interaction with STIM1 CC1 [110,114,115,116,117]. The latter is released upon store-depletion, and then α4 contributes to STIM1 oligomerization and clustering [118]. The STIM1 C-terminal fragment aa 312–387 resolved via NMR unveils a hydrophobic binding pocket for coupling to Orai1 C-terminus [111], with both, the rear portion of CC1 and the CC2 contributing to it (Figure 2d,e).

### 3.2. Orai Proteins

**Figure 3:**
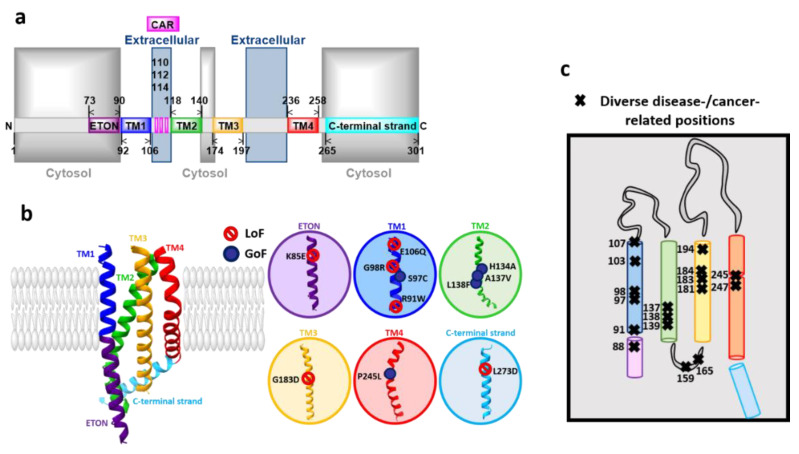
The structural features of the Orai1 channel. (**a**) The scheme shows the full-length human Orai1 channel with highlighted regions and residues that are essential for the Orai1 function. (**b**) The cartoon of one Orai1 subunit with four TM segments along with N- and C- terminal helices are depicted in distinct colors (same as applied within (**a**)). The separated circles of the respective Orai1 subunit regions display the most prominent mutations that are known to lead to either loss of function (red stop sign) or gain of function (blue circle) of the Orai1 channel. (**c**) The scheme of Orai1 subunit with marked residues represent positions linked to diverse diseases or cancer.

All three Orai homologues represent highly Ca^2+^-selective ion channels expressed in the plasma membrane. Currently available crystal and cryo-EM structures from *Drosophila melanogaster* Orai (dOrai) have revealed a hexameric stoichiometry [70,119,120,121]. The high homology of the transmembrane domains (TMs) of dOrai and human Orai1 (hOrai1) suggests that hOrai1 forms a similar hexameric assembly. Each Orai subunit is composed of four transmembrane (TM) domains connected via two extracellular loops and one intracellular loop (Figure 3a). Both, N- and C-termini are located in the cytosol. Among all the three isoforms, the TM domains are highly conserved, whereas the cytosolic strands and connecting loops exhibit major structural differences [66,122,123]. Moreover, we propose an isoform-specific structural difference of the TM2-loop2-TM3 region [124]. While the cytosolic extension of TM2 is longer in Orai3 than in Orai1, the flexible loop2 portion connecting TM2 and TM3 in Orai3 is shorter than in Orai1 [124].

Similar to STIM protein, Orai channels exhibit extensive expression in a diversity of tissues [88,89,90]. Orai1 proteins are in particular highly expressed in immune cells [56,125,126]. Moreover, Orai1 and Orai3 proteins display a wide tissue expression including the heart, brain, kidney, lung, skeletal muscle, and other organs [68,88,127]. Orai2 occurs mainly in the brain and at lower levels in the spleen, lung, and small intestine [48,87,88,128,129]. Besides the expression of Orai isoforms in healthy tissue, they have been found additionally in a variety of different cancer cell types [95,130].

#### Orai Structure

**Figure 4:**
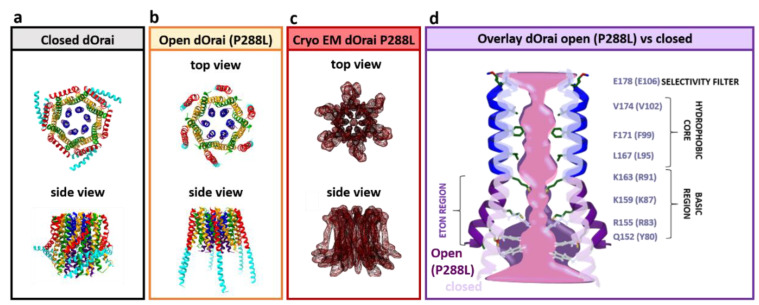
Closed versus open dOrai structure and pore architecture. (**a**–**c**) The top and corresponding side view of the dOrai channel crystal structure of the closed state (**a**), open state (P288L) (PDB ID: 6AKI) (**b**) and cryo EM structure of the open state (P288L) (**c**) are depicted. (**d**) The pore region of the closed state (light-colored TM1 helices) and the corresponding pore profiles are depicted in pink. The structure is overlaid by an open pore structure of dOrai P288L (dark blue and purple TM1 helices) while its pore architecture is depicted in dark purple color.

The hexameric Orai channel complex can be divided into three rings. The Orai pore is composed of six TM1 domains assembled as a ring in the center of the channel complex. It is surrounded by a second concentric ring formed by the TM2 and TM3 and a third ring constituted by the TM4 regions [70,119,120,121] (Figure 4a–c).

Diverse recent reports have demonstrated that several residues within the Orai TM regions keep the entire channel complex in the quiescent state as their point mutation can result in constitutively active channels. They are known as gain-of-function (GoF) mutations [131,132,133,134,135,136,137,138,139]. Besides the structural resolution of the dOrai closed state, GoF mutants are extremely useful for further cryo-EM and crystallographic studies, as they enable to resolve open conformations of the channel. Specifically, the GoF mutants Orai1 H134A (equivalent to dOrai H206A) [70,121,132] and Orai1 P245L (equivalent to dOrai P288L) have been employed for crystallographic studies [119,120] (Figure 4a–c).

The diverse currently available dOrai structures consistently reveal that the TM1 domains extend by an approximately 20 Å long helical region into the cytosol [70] (Figure 3b). In human Orai1, it has been named as the extended TM Orai1 NH_2_-terminal (ETON, aa: 73–90 in hOrai1) region [140]. Furthermore, TM2 and TM3 have been resolved to expand by several helical turns into the cytosol [70].

The quiescent dOrai structure has revealed that the TM4 domain contains a kink formed by P245 in hOrai1 (equivalent to dOrai P288), thus separating the TM4 into two regions, TM4a and TM4b. This proline is fully conserved among the three isoforms. The hinge or the so-called nexus region (aa: 261–265) connects the TM4b domain by a bend to the cytosolic C-terminus (TM4-ext). Moreover, the C-termini of two neighboring subunits form an antiparallel oriented coiled-coil packing and thus, exhibit an overall belt-like structure at the intracellular side of the channel [70,120,135]. Hence, at the structural level, the Orai complex possesses, besides the six-fold, a three-fold symmetry (Figure 3b and Figure 4a–c).

The major conformational differences between the closed and the open dOrai structures have been detected in the basic region of the pore as well as the TM4 domains. All dOrai open structures reveal consistently that the pore region features a 10 Å extension at dOrai K159 residue (equivalent to hOrai1 K87) in contrast to a 6 Å wide pore of the closed dOrai (Figure 4d). Concerning structural resolutions of the TM4-C-terminus segment, the open dOrai crystal and cryo-EM structures exhibit distinct results. The dOrai crystal structures in the active conformation show a fully straightened, the so-called unlatched, conformation of the TM4-C-terminus region extending by ~ 45 Å into the cytosol. Contrarily, the cryo-EM structures of constitutively active dOrai mutants do not show such huge structural changes at the channel periphery [119,120,121,141] and indicate a more latched open Orai conformation (Figure 4a–c).

With respect to the loop regions, the structure of the first extracellular loop has been recently resolved to form an electronegative turret at the pore entrance [121]. The structural resolution of the intracellular and the second extracellular loop, as well as the initial part of the N-terminus, is still unknown.

Currently available dOrai structures represent either a potential inactive or an active state. Nevertheless, these structural studies have only been performed in the absence of STIM1. At the physiological level, Orai channels are activated upon STIM1 binding which induces the conformational changes leading to pore opening. Thus, the ultimate goal is still to resolve the structure of a STIM1-Orai1 complex, which might enable to resolve differences to the currently available open structures of the GoF point mutants.

### 3.3. Activation Mechanisms of the STIM1/Orai Signalling Machinery

**Figure 5:**
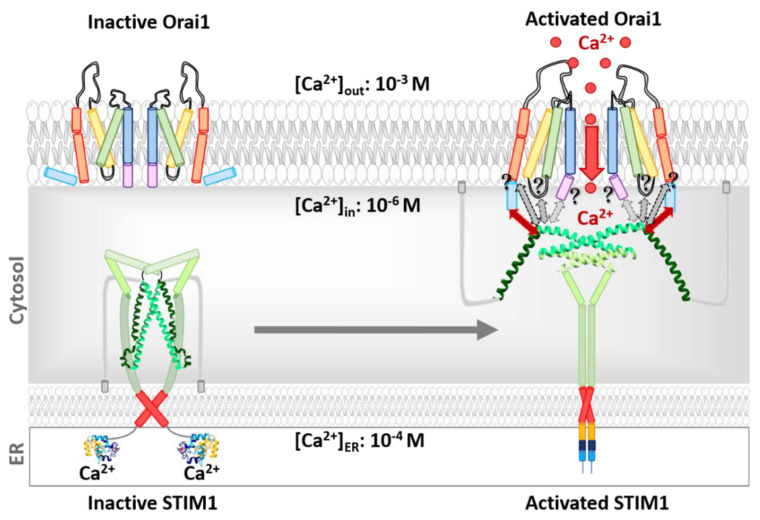
Activation mechanism of the STIM1/Orai signaling machinery. The scheme visualizes STIM1 and Orai1 in the resting state (**left**) and activated state (**right**). Upon STIM1 binding, Orai1 C-terminus is proposed to extend ~45 Å into the cytosol (**right**). The main and indispensable coupling sites for STIM1 represent the Orai1 C-terminus. The hinge plate, loop2, and N-terminus of Orai1 are additionally supposed to interplay with STIM1, either directly or indirectly (as indicated by the double-sided arrows and question marks).

Prior to presenting detailed activation machinery, we provide in this paragraph a short overview of the activation steps of CRAC channels (Figure 5) in lymphocytes and mast cells. Their activation represents a unique, spatially and temporally controlled process that precisely regulates the Ca^2+^ homeostasis via store-operated Ca^2+^ influx. Under resting cell conditions, dimers of the Ca^2+^ sensor protein STIM1 are rather homogenously distributed throughout the ER membrane and exhibit high mobility with a diffusion constant in the range of 0.2 µm^2^/s [142,143,144]. The initial trigger for STIM1/Orai1 activation represents the binding of a ligand to an antigen receptor of immune cells.

This event orchestrates a series of signaling events involving G-proteins or a tyrosine kinase cascade to activate phospholipase C. The latter cleaves phosphatidylinositol 4,5-bisphosphate (PIP_2_) in the plasma membrane, which mediates the production of diacylglycerol (DAG) and inositol 1,4,5-trisphosphate (IP_3_). IP_3_ acts as a secondary messenger and binds to the IP_3_ receptors (IP_3_R) located in the ER membrane (Figure 1). Subsequently, Ca^2+^ is released from the ER stores and STIM1 detects the drop in [Ca^2+^] via its EF hand. To reach its active conformation, it undergoes a conformational change, oligomerizes, and moves into the ER-PM junctions [48,66]. These well-defined and dynamic microdomains where the ER is in close proximity to the plasma membrane provide a platform for stable assembly of the STIM1/Orai1 complex and STIM1-mediated activation of Orai1 [145,146,147,148] (Figure 5). The association of STIM1 and Orai1 is further facilitated by the binding of STIM1 C-terminus to PIP_2_ localized within the PM [67,105]. With the coupling of STIM1 and Orai1 the diffusion of the complex drastically slows with a diffusion constant in the range of 0.03 µm^2^/s [144]. The Ca^2+^ influx through activated Orai1 increases the local [Ca^2+^]_i_ within the micromolar range [149]. The Ca^2+^ ions accumulate in the so-called Ca^2+^ microdomains, that expand upon the clustering of Orai channels [149,150,151]. Upon maximum stimulation, the global [Ca^2+^]_i_ only exceeds by less than 1 μM [150]. This Ca^2+^ influx is critical for the downstream signaling events affecting the transcription factors [152,153,154,155], vesicular fusion proteins [156], other ion channels [157], and particular enzymes [158]. In immune cells, SOCE triggers cytokine production via the nuclear factor of activated T cells (NFAT) and release of inflammatory mediators (histamine and eicosanoids) [155,159,160,161] (Figure 1).

### 3.4. STIM1 Activation

**Figure 6:**
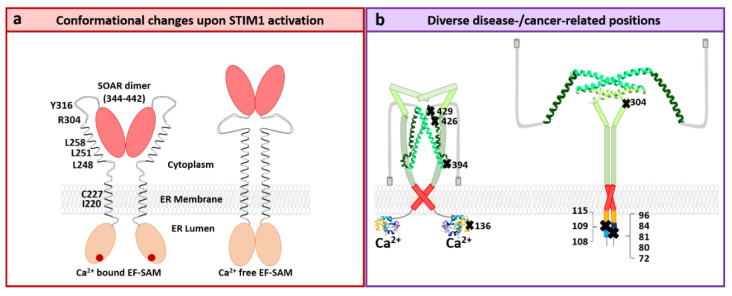
STIM1 activation and diverse positions related to diseases. (**a**) Cartoon representation of STIM1 dimer in the inactive state (left) and activated state (right). In the inactive state, the EF-SAM domains are both loaded with Ca^2+^ ions and do not display any interaction. The transmembrane (TM) helices that connect the N-terminus of STIM1 to the C-terminus in the cytoplasm are spatially separated. The CC1 region displays an “inhibitory helix pocket” with important residues maintaining the STIM1 quiescent state highlighted. SOAR, comprised of residues 344–442, forms intramolecular interactions with CC1. In the activated state (right), the SOAR region decouples from CC1 and is exposed, the EF-SAM domains interact and the transmembrane helices moving closer to another (modified according to Hirve et al. [163]). (**b**) Diverse positions within the STIM1 protein leading to either loss of function (left) or gain of function (right). The highlighted residues represent disease-related positions that can lead to immunodeficiencies, tubular aggregate myopathy, Stormorken syndrome, York platelets syndrome, or even cancer upon mutation.

STIM1 is maintained in the closed state via three structural prerequisites: (1) A Ca^2+^ ion bound to the EF-SAM domain at the N-terminus [75,162]; (2) a particular conformation of STIM1 TM domains [101,163]; and (3) an inhibitory clamp established within the C-terminus [101,118] (Figure 5 and Figure 6a). Store depletion initiates the dissociation of Ca^2+^ from the cEF hand and triggers a conformational change of the EF-SAM region. Exposure of the hydrophobic regions of the EF-SAM domains mediates the oligomerization of close-by EF-SAM regions [75,98,100]. These structural changes initiate signal transduction via the TM domain to the STIM1 C-terminus. The TM domain undergoes conformational rearrangements. In this regard, a recent study has reported that STIM1 TM domains cross each other at a certain angle in the resting state, which decreases upon STIM1 activation and is likely facilitated by three glycines located in the TM region [101]. Moreover, I220 and C227 in the TM domain contribute to the maintenance of the closed state [101]. Another study reported that STIM1 TM helices pair only upon STIM1 activation [163]. Thereby, they overcome the STIM1 C-terminal intramolecular clamp established by CC1 (L248, L251, L258) and CC3 (L416, L423), which is released upon store-depletion. This event is accompanied by the exposure of the SOAR region, which establishes an interaction with Orai1 [101,118]. Moreover, a STIM1 homomerization domain partly overlapping with CC3 promotes STIM1 oligomerization [106,118] (Figure 5 and Figure 6a). Subsequent to STIM1-Orai1 coupling, Orai1 is activated.

Moreover, STIM1 clustering in the ER-PM junctions is facilitated by a lysine-rich region at the very end of the STIM1 C-terminus. It interacts with the plasma membrane PIP_2_ and stabilizes the STIM1 puncta (Figure 5). Thus, Orai1 originally diffusely distributed in the plasma membrane is recruited into the areas of the STIM1 aggregates [67,105].

### 3.5. STIM1-Orai1 Coupling

The STIM1-Orai1 coupling is established via essential domains that are described in the following (Figure 5).

#### 3.5.1. STIM1 Domains Coupling to Orai1

As previously described, the CAD or SOAR domains in STIM1 C-terminus represent the direct interaction sites for Orai1 C-terminus, that induce Orai1 activation. As shown via the minimal STIM1 C-terminal fragments (OASF, SOAR, CAD, or Ccb9), mainly incorporating CC2 and extended CC3 [106] domains are sufficient to trigger Orai1 activation [80,106,107,108,140] (Figure 2a). It is currently clear that Orai1 C-terminus is the main and strongest coupling site for STIM1 C-terminus. The structural resolution of SOAP, a complex of the key interaction sites Orai1 and STIM1 C-termini (STIM1 aa: 312–387; hOrai1 aa: 272–292), is perfectly in line with the functional characterization of these critical sites [111]. The positions within a STIM1 dimer most critical for coupling to two Orai1 C-termini are L347, L351 in CC2 and Y362, L373, and A376 in the second CC2 domain [111,164]. The positively charged residues K382, K384, K385, and K386 further stabilize the STIM1-Orai1 coupling [111] (Figure 2d,e and Figure 5).

Whether additional STIM1 C-terminal sites couple to either Orai1 N-terminus or the loop2 is still under debate. There is evidence that SOAR α2 plays a role in the Orai1 coupling as well as in SOCE activation [165]. Potential direct interaction of the non-conserved STIM1 residue F394 in α2 has been proposed either between the Orai1 N-terminus or the so-called hinge plate [113,165]. This Orai1 hinge plate is located between the two leucines within the cytosolic helical regions of TM3 (L174) and TM4 (L261) [113,165]. Additionally, we recently discovered that a close proximity of STIM1 α3 and Orai1 TM3 contributes to Orai1 gating [1] (Figure 5).

In summary, residues within STIM1 crucial for interaction with Orai1 C-terminus are resolved. Whether additional sites within STIM1 C-terminus are involved in the interaction with other cytosolic structures of Orai1 still requires further investigation.

#### 3.5.2. Orai1 Domains Coupling to STIM1

Within the Orai1 C-terminus, the main coupling site for STIM1 C-terminus, the residues L273 and L276 are crucial for interaction with STIM1 [74,78,166,167], as their mutation to hydrophilic amino acids such as serine or aspartate (e.g., L273S/D) fully abolish the communication with STIM1. The NMR SOAP structure has revealed that R281, L286, and R289 in Orai1 C-terminus additionally contribute to STIM1 coupling [111] (Figure 5).

Moreover, the communication of STIM1 with the Orai1 hinge plate, composed of L174 in TM3 and L261 in TM4, is crucial for CRAC channel activation. Their single point mutation can lead to reduced or impaired Orai1 channel function, likely because of a disturbed communication of the two hydrophobic leucines [113,135]. Furthermore, the hinge region LVSHK, connecting TM4 to the C-terminus and located next to the hinge plate, determines STIM1 coupling, as multiple point mutations (Orai1 ANSGA), which induce constitutive activity, further reduce STIM1 binding [135]. Furthermore, it has been shown that the region around E173 in Orai1, which is close-by to the hinge plate, is indispensable for STIM1-mediated Orai1 gating [1] (Figure 5).

Another important region for STIM1-Orai1 coupling and subsequent Orai1 gating close-by to the hinge region represents the loop2-TM3 segment aa: 160–170. Cysteine crosslinking of STIM1 L402C with Orai1 E166C allowed Orai1 activation, while the break of disulfide bonds abolished their currents [1] (Figure 5).

Besides Orai1 C-terminus and loop2, Orai1 N-terminus plays an essential role in communication with STIM1, either directly or allosterically. Deletion of or single point mutation within Orai1 N-terminus can abolish STIM1-mediated activation. We discovered that a deletion of the first 76 amino acids of Orai1 leads to loss of function [140]. Moreover, the N-terminal residues L74, W76, R83, K85, K87 possess a critical role in STIM1-mediated activation, as their mutation can abolish Orai1 activation [140]. Additionally, an intact N-terminus is essential for STIM1-induced maintenance of authentic CRAC channel hallmarks [168,169]. Biochemical assays revealed an interaction of STIM1 C- and Orai1 N-terminal fragments, which was reduced upon deletion or single point mutation within the N-terminus [140,169]. Nevertheless, whether STIM1 C-terminus directly binds to the N-terminus in full-length Orai1 remains elusive [107,140]. We recently discovered that a permissive communication of the Orai1 N-terminus with the loop2 region is critical for the maintenance of the activation of Orai1 [124]. There is indisputable evidence that an intact N-terminus and loop2 are indispensable for normal CRAC channel function. Nevertheless, whether either Orai1 N-terminus, the loop2, or a complex of both, represent the STIM1 coupling site still requires further investigation (Figure 5).

In summary, the main coupling sites for STIM1-Orai1 interaction represent STIM1 and Orai1 C-termini. Additionally, Orai1 N-terminus, loop2, and the hinge plate are essential for direct or indirect communication with STIM1. The hinge plate seems to control the signal transmission from the STIM1-Orai1 coupling site at the channel periphery to the Orai1 pore region.

### 3.6. Activation of the Orai1 Ion Channel

Activation of the Orai1 channel is initiated via STIM1 coupling to the Orai1 C-terminus [78,134,170]. This signal is subsequently transmitted from the channel periphery across all four TM domains finally to the pore [66,123].

A comparison of the crystal structures of the closed dOrai and the open GoF-dOrai mutants suggests two major structural alterations within the channel complex upon Orai activation: (1) Orai1 pore widening within the basic region by approximately 4 Å and (2) conformational changes at the Orai complex periphery (Figure 4a–c). However, it is still controversial to which extent structural alterations occur along the TM4-C-terminus region [70,119,120,141]. A straightening of the TM4-C-terminus region is assumed to be energetically unfavorable under physiological conditions. Recent MD simulations have revealed that pore opening involves a twist-to-open gating motion. At the extracellular side, all subunits have been supposed to rotate counterclockwise. At the intracellular side, every second subunit moves outward, while the other three subunits rotate clockwise [171].

Independent to which extent conformational changes take place at the outmost side of the channel complex, the site-directed mutagenesis revealed that the kink at P245L within TM4 and the nexus region establishing the bent connection between TM4 and the C-terminus determine the closed and open state of the Orai1 channel. In support, mutation of these regions can lead to constitutively active Orai1 channels [134,135,139] (Figure 4a–c).

An arsenal of gain- and loss-of-function mutations located within all four TM domains suggested that Orai1 pore opening involves a global conformational change of the entire channel complex [133,134,136,138,172,173,174]. We recently demonstrated via a library of double mutants systematically combining one GoF- with one LoF-mutation in the distinct TM domains, that Orai1 pore opening indispensably requires structural changes within the entire channel complex. For this purpose, a series of control points in the middle and cytosolic part of all TM domains must have an opening-permitting conformation and enable clearance for the pore opening [175].

STIM1-induced conformational changes across the entire channel complex are supposed to cause a rotation of the hydrophobic region within TM1 [133], specifically of the positions G98 and F99 [133]. Moreover, Frischauf et al. [132] have reported structural alterations within the basic region, in particular of R91.

Overall, STIM1-mediated Orai1 activation induces a wave of interdependent TM domain motions across the entire channel complex, which requires clearance of a series of gating checkpoints within the Orai1 channel.

### 3.7. The Orai1 Pore

The pore region of the Orai1 channel can be divided into four regions. At the extracellular side, the pore consists of three aspartates (D110, D112, and D114) of each subunit which constitute the Ca^2+^-accumulating region (CAR). It initiates Ca^2+^ permeation due to the attraction of Ca^2+^ ions [176]. Subsequent to the CAR region the pore contains the selectivity filter, the narrowest part with a diameter of ~3.8 Å therein [69,70]. It is built by a ring of six glutamates (E106) [177] (Figure 4d). In support, the Orai1 E106D mutant [69] exhibits loss of Ca^2+^ selectivity, whereas Orai1 E106Q [178] represents a loss-of-function pore mutant. The selectivity filter is followed by a hydrophobic area containing V102, F99, and L95 [70], which function together with the selectivity filter as the Orai1 gate. Moreover, this hydrophobic cavity controls Ca^2+^ selectivity together with STIM1 via rotation of the region [133,179]. STIM1 coupling has been demonstrated to move F99 out and G98 into the pore to allow Ca^2+^ permeation [180]. At the cytosolic side, the pore region is formed by a basic segment [140] specifically composed of positively charged residues R91, K85, and R83 within the ETON region (Figure 4d). Structural studies have suggested that these positively charged side chains are neutralized by anionic aggregates, which impair ion conduction in the closed channel either via steric occlusion or electrostatic repulsion [70]. Moreover, anions have been found to bind in the open dOrai P288L structure to the basic region, which is probably necessary for Ca^2+^ permeation [119]. Indeed, their mutation to residues with neutral or oppositely charged side chains has reduced or abolished the activity of Orai1 [119]. MD simulations have further revealed that the basic residues rather impact pore hydration [181].

### 3.8. Unique Biophysical Properties of CRAC Channels

The unique biophysical properties of CRAC channels described in detail in Krizova et al. [168] include extremely high Ca^2+^ ion selectivity, exceptionally small unitary conductance, fast Ca^2+^-dependent inactivation (FCDI), and an increase in currents upon the exchange from a Ca^2+^-containing to a Na^+^-containing divalent free solution.

The typical CRAC channel inward rectifying currents exhibit a reversal potential of ~+50 mV [71,182]. The high Ca^2+^ selectivity has been suggested to be established via ion-pore and ion–ion interactions [183]. Interestingly, the permeability of CRAC channels for Ca^2+^ ions is 1000 times higher than that of monovalent ions [184,185,186,187,188,189,190]. The presence of Ca^2+^ in a divalent free solution inhibits monovalent ion permeation [185,186,187,188,189,190]. In the absence of Ca^2+^, monovalent cations such as Na^+^, Li^+^, and K^+^ permeate across Orai1-activated channel via STIM1 [185,186,187,188,189,190]. The narrow pore diameter of the Orai1 channel strictly limits the permeation of Cs^+^ or larger ions [131,150,177,179,180,191]. The trivalent ions such as La^3+^ or Gd^3+^ block the Orai1 pore completely [48,150]. Many direct mutagenesis studies demonstrate that the permeability of Orai1 channels is highly modulated by STIM1 [48,66,123,131,177,180,191,192], as diverse constitutively active Orai1 mutants are less selective in the absence of STIM1 and restore Ca^2+^ selectivity in the presence of STIM1 [131]. The exceptionally small unitary conductance has been estimated via noise analysis to be in the range of 10 fS [150,193] and likely underlies the very narrow pore diameter [194,195]. Up until now, because of this distinctive feature, no single CRAC channel patch-clamp experiment has been feasible [150,196].

Enhancement of cytosolic Ca^2+^ levels via CRAC channels is strictly limited via the Ca^2+^-dependent inactivation functioning as a Ca^2+^-dependent feedback mechanism. There are two kinds of CDI known, the fast (FCDI) and the slow (SCDI) [197,198]. Whereas the FCDI is accomplished within tens of milliseconds and is recorded during a hyperpolarizing voltage step, the SCDI requires minutes for full completion. The SCDI is detected upon the applied repetitive voltage ramps.

Another outstanding biophysical feature of CRAC channels is the increase of currents when the Ca^2+^-containing solution is exchanged by a Na^+^-containing divalent free one. This unique property is assumed to correlate with the degree of FCDI. Indeed, CRAC channels exhibit FCDI in a Ca^2+^-containing solution, while no FCDI is detectable in a Na^+^-containing divalent free solution, in accordance with the steady-state current size in Ca^2+^- and Na^+^-containing solutions.

### 3.9. Pharmacology of CRAC Channels

The identification of STIM and Orai molecules and their association with a variety of diseases boosted the search for small molecule modulators of CRAC channels. Currently available inhibitors have helped improve our understanding of the CRAC channel machinery. Their targets of action include one of the different steps in the STIM1/Orai1-activation cascade. Nevertheless, most of those have not reached clinical trials yet, because of the low selectivity and high toxicity [199,200,201].

Well-known general Ca^2+^ ion channel pore blockers represent the lanthanides such as La^3+^ (lanthanum) and Gd^3+^ (gadolinium), which act at submicromolar concentrations. It is worth noting, that especially CRAC channels can be blocked by lanthanides at very low concentrations, in contrast to a diversity of other Ca^2+^ ion channels, which require higher amounts for inhibition [184,202].

SKF-96365, a compound that belongs to the imidazoles, is one of the first elucidated CRAC channel blockers, however, it possesses low selectivity and its mechanism of action is unknown [200,203].

2-Aminoethyldiphenyl borate (2-APB), a drug that pertains to the diphenylboronate compounds, is non-selective, but the best-characterized blocker of CRAC channels. It exhibits complex pharmacology and interferes with CRAC channel currents in a biphasic manner. While low concentrations (1–10 µM) of 2-APB enhance CRAC channel activity, higher concentrations (20–100 µM) lead to a transient activation followed by inhibition [204,205,206]. Among the Orai isoforms, 2-APB acts in an isoform-specific manner. In contrast to Orai1 currents, which respond with the above described biphasic behavior, Orai3 currents are strongly enhanced by 2-APB and become non-selective [207,208]. 2-APB acts not only on the Orai1 channel but has been further identified to interfere with several steps of the CRAC channel activation cascade including STIM1 oligomerization, STIM1 conformational change, STIM1-Orai1 coupling, and on the Orai channel itself [71,209,210,211]. Moreover, 2-APB influences the activation of other ion channels, such as some TRPVs and the IP_3_ receptor [212,213]. Within recent years, two 2-APB analogues (DPB162-AE, DPB163-AE) have been identified, which exhibit higher specificity and potency than 2-APB in terms of CRAC channel inhibition [204,205,214,215]. 2-APB has been further reported to enhance STIM2-mediated Orai1 currents in a store-independent manner [216].

Furthermore, the pyrazole compounds, BTP2 [217,218], Pyr6, and Pyr10 [219], are useful tools to inhibit CRAC channels. BTP2 displays a 30-fold higher selectivity for store-operated Ca^2+^ channels than voltage-gated Ca^2+^ ion channels [218]. Aside from its action on CRAC channels, BTP_2_ has been reported to activate TRPM4 and to block TRPC3 and TRPC5 currents [220,221]. Interestingly, Pyr6 displays a higher potency to inhibit CRAC channels, while Pyr10 is selective on TRPC3-mediated Ca^2+^ currents [219].

The GSK blockers, GSK-5498A, GSK-7975A, and GSK-5503A represent very selective blockers as they act on almost no other ion channels [222,223] except TRPV6 channels. GSK-7975A has been supposed to alter the pore geometry of Orai channels [224].

Synta66 is a selective CRAC channel blocker [158,225]. We recently demonstrated that Synta66 binding requires residues at the extracellular side of Orai1 close to TM1, TM3 and the extracellular loop regions. Indeed, Orai1 mutants reducing the Ca^2+^ selectivity interfere with the action of Synta66 [226]. Concerning the action of GSK-5503A and Synta66, it has been recently shown that the different Orai isoforms show distinct responses to the application of these inhibitors [214].

An inhibitor of the myosin light chain kinase, ML-9, reversibly inhibits CRAC currents. It has been identified to interfere with STIM1 multimerization, however, the site of action remains unknown [227].

Diethylstilbestrol (DES) is another inhibitor useful to study CRAC channel function. It is assumed to act on the CRAC channel pore geometry. However, as it additionally activates estrogen receptors, it is not suitable for clinical use [228,229].

Linoleic acid, a polyunsaturated fatty acid, interferes with STIM1 oligomerization and thus blocks STIM1-mediated Orai1 currents. The mechanism of action, however, is still elusive [230].

Highly selective blockers that have reached the stage of clinical trials represent carboxyamidotriazole (CAI), RO2959, CM2489, and CM4620. Other Orai1 specific blockers represent a series of 1-phenyl-3-(1-phenylethyl)urea derivatives. Calcimedica suggests that the binding pocket of CM2489 and CM4620 is composed of certain residues in TM2, TM3, and TM4, which have also been identified to be involved in keeping Orai1 in the closed state [199,200,231]. It is worth noting that the CRAC channel blocker CM4620 represents a promising candidate to treat patients suffering from severe respiratory dysfunctions including COVID-19 pneumonia [232].

In summary, a series of CRAC channel blockers is available (Table 1). Despite many of them show low selectivity and their mechanisms of action are unknown, they represent useful tools to study the ion channel structure/function relationships and STIM1/Orai1-mediated signaling steps [192,233].

## 4. Pathophysiological Functions of STIM1 and Orai1

The correct function of STIM and Orai proteins is indispensable to guarantee healthy processes in the human body. Currently, a series of gain- and loss-of-function mutations are known that can lead to severe diseases. Moreover, an altered expression level of these proteins can impact the normal processes. The diverse pathophysiological roles of the CRAC channel proteins are summarized in Table 2.

### 4.1. STIM1 and Diseases

Among the currently known disease-related STIM1 mutants, many of them are linked to severe combined immunodeficiency (SCID) [68], Stormorken syndrome (STRMK) [237], and tubular aggregate myopathy (TAM) [238,239,240]. Generally, dominant STIM1 gain-of-function (GoF) mutants have been associated with STRMK and TAM, while recessive loss-of-function (LoF) mutations can lead to SCID [63]. STRMK and TAM represent distinct spectra of the same multisystemic disease which has been discovered by muscle weakness, ichthyosis, thrombocytopenia, miosis, short stature, dyslexia, myalgia, and hyposplenism [241]. Overall, 47 TAM/STRMK families have been reported with related histological, clinical, and genetic data [12,63,237,241,242,243].

Prominent STIM1 mutations associated with TAM, that cause constitutive activity represent for instance H72Q, D84G, H109N, H109R located in the highly conserved region of EF hand motif [238]. These mutations reduce the Ca^2+^-binding affinity of STIM1, thus, explaining their constitutive activity [238,239,244]. STIM1 R304W containing a gain-of-function mutation in its C-terminus has been associated with the Stormorken syndrome [139] (Figure 6). Another mutation R429C, which is situated in STIM1 C-terminus, impairs coupling to Orai1 and has been connected to combined immune deficiencies [245] (Figure 6b). Other disease-related loss-of-function mutants include for instance STIM1 R426L, associated with enamel maturation [246] or STIM1 E136X, which truncates STIM1 at the beginning of the SAM domain, and has been associated with severe immunodeficiency [247].

Lack of STIM1 expression has been reported to cause immunodeficiencies including severe bacterial, viral, or fungal infections and thus, repeated episodes of pneumonia, meningitis, or gastroenteritis [63]. Additionally, STIM1-deficient patients exhibit symptoms of autoimmunity, hemolytic anemia, thrombocytopenia, or anhidrotic ectodermal dysplasia (EDA) [63]. The development of diverse cancer types such as glioblastoma, breast cancer, prostate cancer, and hepatocellular carcinoma has been linked to an upregulated expression of STIM1 proteins [32].

### 4.2. Orai1 and Diseases

Diverse disease-related Orai1 mutants are known to be responsible for the development of SCID [68], Stormorken-like syndrome [139], TAM [248], autoimmunity, and ectodermal dysplasia (EDA) [56,91,249]. Orai1 mutations leading to constitutive activity and associated with TAM represent S97C [248], G98S [136], V107M located in TM1, L138F situated in TM2 and T184M in TM3 [136,137]. The prominent GoF mutant P245L located in TM4 is connected to the Stormorken-like syndrome. The recessive Orai1 LoF mutations in TM1, R91W, G98R, and A103E, are linked to immunodeficiencies [68,91,249]. Additionally, Orai1 V181SfsX8 and L194P, representing LoF mutations in TM3, lead to autoimmunity and ectodermal dysplasia [91,249] (Figure 3c).

### 4.3. CRAC Channels and Cancer

CRAC channel activity is indispensable for healthy processes in the cell. Nevertheless, over the last decade, indisputable evidence arose that the CRAC channel components are involved in the development and growth of cancer cells.

Diverse mutations within both, STIM1 and Orai1, have been reported to be connected to different types of cancer, like lung adenocarcinoma (STIM1 A79T, E87Q, W350L, G446C/V), glioblastoma (Orai1 G183D, STIM1 S116N), uterine carcinoma (Orai1 S159L), colorectal tumor (Orai1 A137V), stomach carcinoma (Orai1 M139V), skin melanoma (STIM1 T517I, S521L), neck carcinoma (Orai1 G247S) [132,250] (Table 2). In addition to these mutations, the upregulation of CRAC channels can lead to cancer cell progression.

Expression of STIM1 and Orai proteins have been verified for instance in breast cancer, lung cancer, glioma carcinoma, colorectal cancer, cervical cancer, prostate cancer, hepatocellular carcinoma, gastric cancer, and others [32,279,280,281]. Among the patients suffering from colorectal cancer [260,261], gastric cancer [262], cervical cancer [263,264], a correlation between STIM1 and/or Orai1 expression levels and poor prognosis with fast metastatic progression has been detected. Despite the expression levels of the CRAC channel components have been extensively studied in a variety of cancer cell types [32], the molecular mechanisms that govern their overexpression are only emerging to get resolved.

In the following, we summarize the current knowledge on CRAC channel components in certain cancer types. We specifically focus on breast, colon, and prostate cancer (Section 4.3.1, Section 4.3.2 and Section 4.3.3) as their development and growth have been reported to be triggered by a co-regulation of CRAC channels with Ca^2+^-activated K^+^ ion channels as discussed later in the review (Section 9). Moreover, we briefly tough the relevance of STIM and Orai proteins in other carcinomas. Additionally, the current knowledge on the role of CRAC channel components is summarized in Table 2 and in the following excellent reviews [32,280,282,283].

#### 4.3.1. Breast Cancer

A combination of knockdown and rescue strategies in the breast cancer cell line MDA-MB231 has revealed that STIM1 and Orai1 play a major role in breast cancer cell migration [284]. The knockdown of STIM1 or Orai1 led to drastically reduced metastasis [284]. Besides Orai1, Orai3 seems to play a role in breast cancer development [59,258]. Orai3 expression is upregulated in ER^+^ MCF-7 and T47D breast cancer cell lines [59,258,285]. Orai3-mediating SOCE has been demonstrated to trigger proliferation and invasion of ER^+^ MCF-7 cells and selectively control the estrogen receptor-α (ERα) [285]. Interestingly, also STIM1-independent Orai1 activation mechanisms have been detected in breast cancer cells. In the breast cancer cell line MCF-7, the accessory protein secretory pathway Ca^2+^-ATPase 2 (SPCA2) [254], conventionally operating as a Golgi Ca^2+^ pump, has been identified to directly interact with the Orai1 channel at the cell surface [254]. Co-IP and pull-down experiments have shown that SPCA2 is trafficking to the PM, where it induces constitutive Orai1 activation. The latter is accomplished without Ca^2+^ store depletion and in a STIM1-independent manner and has been therefore named as SICE (store-independent Ca^2+^ entry) [254,255]. SICE due to a co-expression of SPCA2 and Orai1 has been reported to drive breast cancer cell growth (Table 2). Moreover, a co-regulation of SK3 with Orai1 in a STIM1-independent manner has been reported to control breast cancer cell development [34,36,271] (Table 2), which is reviewed in detail in Section 9.

Overall, Orai1-mediated SOCE plays a crucial role in breast cancer cell metastasis, while Orai3 is involved in proliferation and cell survival.

#### 4.3.2. Prostate Cancer

Prostate cancer develops rather because of resistance to apoptosis, than because of enhanced proliferation. In the early state, prostate cancer depends on the androgens, while later it develops into an androgen-independent type of cancer, which is more aggressive. This apoptotic resistance of androgen-independent prostate cancer cells has been shown to correlate with abrogated SOCE [62]. Specifically, the expression level of Orai1 is downregulated, hence, SOCE is diminished when androgen levels are lowered [62]. In support, Orai1 overexpression has restored SOCE and has induced a comparable apoptotic rate to androgen-dependent cells [62]. Hence, it has been supposed that androgens are involved in the regulation of Orai1 expression. Indeed, androgen contact sites have been detected on the Orai1 promoter [62]. Prostate cancer cells in the more aggressive state take advantage of the reduced SOCE, which makes them resistant to apoptosis. In contrast, breast cancer cells profit from increased Ca^2+^ entry to induce cell survival and migration [284]. These two opposite scenarios driving cancer cell growth are still puzzling. A potential explanation might be that distinct downstream Ca^2+^ signaling cascades lead to distinct activation signals. However, this hypothesis still requires further proof.

It has been demonstrated that all three Orai isoforms play a role in cell cycle progression and proliferation through the regulation of cytosolic Ca^2+^ levels [256]. In particular, the expression level of Orai3 in prostate cancer biopsies is upregulated [256], which leads to a reduction in SOCE and enhanced Ca^2+^ influx mediated by arachidonic acid (AA)-activated ARC channels [256]. Contrarily, another study has reported that Orai3 expression is downregulated in prostate cancer cells [259] (Table 2). Altered 2-APB modulation of store-operated currents and knock-down experiments in LNCaP cells have suggested that because of the downregulation of Orai3 expression, heteromeric Orai1/Orai3 channel formation is more frequent than in healthy prostate cells [259].

Altogether, there is emerging evidence that Orai proteins contribute to prostate cancer growth and development, however, the above described discrepancies require further investigation. Moreover, the expression levels of STIM1 and Orai1 and other homologs are found to be differentially regulated based on the prostate cancer stage. Potentially, the controversial results might be detected because of the heterogeneous nature of prostate cancer [286].

#### 4.3.3. Colon Cancer

Colon cancer cells contrary to healthy colon cells have been reported to exhibit enhanced store-operated Ca^2+^ entry. Indeed, a correlation of increased expression of STIM1 and enhanced tumor size, tumor invasion and metastasis has been shown for colorectal cancer [261,287]. It has been demonstrated that activation of STIM1 and Orai1 is required to induce EGF-mediated activation of proinflammatory and prometastatic gene cyclooxygenase (COX-2). Moreover, prostaglandin E2 (PGE2) secretion is increased in colon cancer cells [261]. Another study [287] further demonstrated that enhanced Orai1 and decreased STIM2 expression drive colon cancer cell development. Recently, Zuccolo et al. [288] reported that STIM and Orai proteins contribute to constitutive Ca^2+^ influx in primary cultures of human colorectal carcinoma cells. In this context, the ER Ca^2+^ levels are reduced. While these constitutive currents could be abrogated pharmacologically, proliferation and migration of colorectal carcinoma cells remained unaffected. In addition, TRPC1 and SK3 have been reported to play a role in the development of constitutive Ca^2+^ entry in and progression of colon cancer cells [272,287].

#### 4.3.4. Other Cancer Types

In this section, we provide an overview of the knowledge of the relevance of STIM and Orai proteins in other cancer cell types such as glioblastoma, melanoma, and cervical cancer.

STIM1 and Orai1 play a significant role in human glioblastoma or glioblastoma multiforme (GBM) cells, a form of aggressive brain malignant tumor which originates from glial cells and astrocytes. In glial cells, isolated from tumor biopsies, the level of Orai1 expression is upregulated, while the level of STIM1 seems to be comparable to healthy cells. The enhanced expression of Orai1 leads to an increased SOCE in GBM cells. Surprisingly, knockdown studies of either STIM1 or Orai1 reveal decreased invasion and cell proliferation [289]. Interestingly, another report revealed that both STIM1 and Orai1 are essential for the survival and proliferation of glioblastoma cell lines [289,290]. Zhu et al. [291] have reported that Orai1 expression is elevated which is associated with an enhanced invasion of GBM cells (Table 2).

In melanoma cells, STIM1 and Orai1 have been reported to play a role in migration. Indeed, knockdown of STIM1 and Orai1 reduced the migration of melanoma cells [292]. Furthermore, based on in vivo experiments, STIM2 is assumed to play a role in tumor invasion and metastasis of melanoma [61]. However, the role of STIM2 in melanoma cells has remained less understood.

The expression of STIM1 is elevated in cervical tumors and has been linked to increased metastasis and poor survival [263]. Moreover, Orai1 has been demonstrated to be upregulated in cervical cancer, but not in related normal cells [264].

In renal cancer types, the expression of Orai1 has been reported to be enhanced compared to normal tissues and to drive invasion and cell proliferation [251].

Moreover, STIM1 and Orai3 play a prominent role in lung adenocarcinoma, where they modulate proliferation [293] and apoptosis [294], respectively. In pancreatic adenocarcinoma, upregulated STIM1 and Orai1 control cell survival [32].

Overall, STIM and Orai homologues can be determinants for cancer cell development, however, because of their complex and divergent role, detailed studies are still required to understand their mode of action in cancer cell growth.

## 5. Ca^2+^-Activated K^+^ Channels

Ca^2+^ ions entering the cell are a crucial source for the activation of a diversity of Ca^2+^-sensing proteins. Among the latter, we aim to focus on the structure/function relationship of Ca^2+^-activated potassium ion channels, as they have been reported to interplay with the CRAC channel.

The Ca^2+^-activated K^+^ (K_Ca_) channels can be grouped into three categories: large (BK, K_Ca_ 1.1), intermediate (SK4/IK/K_Ca_3.1), and small (SK1, SK2, SK3/K_Ca_2.1, K_Ca_2.2, K_Ca_2.3) conductance ion channels. They possess a unique feature to connect intracellular Ca^2+^ signals to cell excitability. K_Ca_ channels are widely expressed in the neurons of the central nervous system (CNS), where they are involved in the control of excitability, synaptic signal transduction, and firing pattern. In non-excitable cells, K_Ca_ channels organize K^+^ homeostasis and cell volume. Additionally, they trigger hormone secretion and the release of neurotransmitters. The plethora of functions of K_Ca_ channels reflects their imperative in living organisms. Defective working mechanisms or overexpression of K_Ca_ channels have been associated with neuronal disease [275,295] and many cancer phenotypes [72,296,297,298,299].

BK channels define the membrane potential and possess a very high single-channel conductance of ~100–300 pS. They are activated by both, voltage and enhanced cytosolic Ca^2+^ levels. The single-channel conductance of SK4 channels is in the range of 20–85 pS, while that of SK1–3 channels exhibit 4–14 pS. Small and intermediate K_Ca_ channels activate at low intracellular Ca^2+^ concentration (300 nM) in a voltage-independent manner. Despite BK and SK channels show a rather low homology, both are regulated by Ca^2+^. Nevertheless, their gating mechanism is completely distinct. While BK channels are directly gated by Ca^2+^ ions, the activation of SK channels is triggered upon Ca^2+^ binding to calmodulin (CaM), which is constitutively bound to SK channels. In the following, we will especially highlight the activation mechanisms of SK channels, as in particular SK3 plays a significant role in the interplay with STIM1 and Orai1 [300,301,302,303].

### 5.1. SK Channels

**Figure 7:**
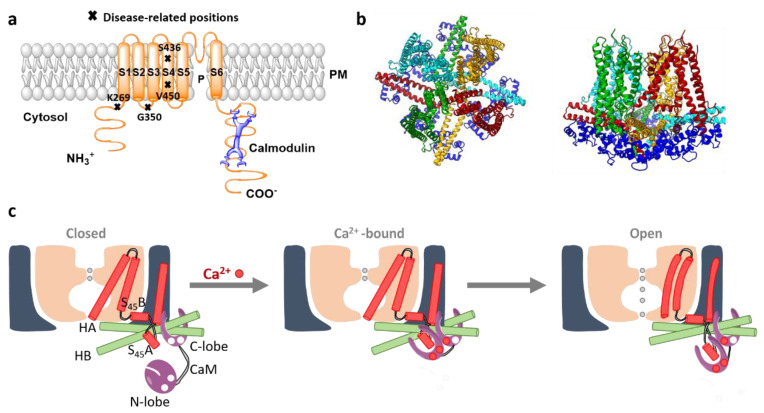
The structure and activation mechanism of SK channels. (**a**) The proposed structure of the SK channel with constitutively bound CaM. P represents the pore region of the channel. Moreover, residues that have been associated with the rare developmental disorder, the Zimmermann–Laband Syndrome, are highlighted. (**b**) The top and side view of SK4 tetramer (PDB ID 6CNM; 4 subunits colored distinct in red, yellow, light blue, green) bound to 4 CaM (dark blue). (**c**) The scheme depicts the stepwise gating mechanism of the SK channel. Under the Ca^2+^ free conditions (**left panel**), the SK channel remains closed. The C-lobe of CaM is constitutively associated to the channel, whereas the CaM N-lobe possesses diverse conformations due to a high level of flexibility and almost no present interaction to the channel. The binding pocket of CaM N-lobe at this stage stays closed. In the presence of Ca^2+^, the ions bind to the CaM N-lobe, which structurally rearranges into a more open conformation (**middle panel**). The latter rearrangement allows the interaction of CaM N-lobe with the S_45_A helix. In the following, the N-lobe pulls the S_45_A toward the cytosol, displacing S_45_B away from the pore axis (**right panel**). Subsequently, the S6 helical bundle expands and potentially opens the pore. Modified from [304].

SK channel complexes possess a tetrameric stoichiometry with each of the four subunits composed of six transmembrane (S1–S6) domains. Thus, they resemble the overall structure of voltage-gated (Kv) K^+^ channels, whereas the voltage sensor S4 is absent. Both, N- and C-termini of SK channels are situated in the cytosol. The pore region of SK channels is formed by a re-entrant loop between the S5 and S6 domains [300] (Figure 7a).

Recently, two cryo-EM structures of the SK4-CaM complex in the closed and open conformation with a resolution of 3.4 and 3.5Å [304], respectively, have been reported. These structures confirm that four SK4 subunits assemble in a four-fold-symmetric tetramer which is ~95-Å long and 120-Å wide [304]. The pore region formed by the re-entrant loop between S5 and S6 domains is surrounded by S1 and S4 helices. The topology of SK channels resembles that of BK channels, however, there are two essential differences in the length of S1 and S2 helices and the structure of the S4-S5 linker. In the SK channel, the S1 and S2 expand to the cytosol and are much longer (60 Å) than those of the BK channels. The S4-S5 linker in the SK channel comprises two α-helices, S_45_A and S_45_B, whereas it forms a short turn in the BK channel. The structure of the S4-S5 linker is assumed to be responsible to transfer Ca^2+^ sensitivity to the SK channel gate mediated by CaM (Figure 7).

At the C-terminal end of S6, two helices H_A_ and H_B_ positioned in parallel to the membrane plane were resolved. The peripheral ends of H_A_ and H_B_ of each SK4 subunit represent the binding site for CaM C-lobes. Four CaMs bind to one SK channel tetramer. H_B_ is still followed by another helix, H_C_, which forms a coiled-coil region positioned in the center of the complex. This region is essential for channel assembly and trafficking [304] (Figure 7).

While the structure–function relationship of all SK channels is comparable, they exhibit distinct expression patterns in specific cell types. SK1–3 channels are found in different kinds of cells including neurons, smooth muscle, and sensory cells [305]. The SK4 channel is mainly expressed in the epithelial cells [296].

### 5.2. Activation Mechanism of a Human SK-Calmodulin Channel Complex

SK channel gating is accomplished by submicromolar changes in cytosolic Ca^2+^ levels (K_D_ = 0.5 µM) [306]. Ca^2+^-dependent regulation of K_Ca_ channels is established via constitutively bound calmodulin (CaM) [302] to the calmodulin-binding domain (CaMBD) at the C-terminus. The first indications of K_Ca2+_ activation via CaM have been obtained upon CaM binding to partially purified K_Ca_ channels from the kidney. Furthermore, SK2 channel deletion mutants have unraveled the proximal C-terminus as the CaM-binding domain, CaMBD. GST fusion protein experiments have revealed that CaM was efficiently bound to the CaMBD, both, in the absence as well as the presence of Ca^2+^ [307,308].

CaM binding to the C-terminus of SK channels has been further verified via the structural resolution of a complex of a C-terminal fragment of SK2 channels together with Ca^2+^/CaM. This structure shows an elongated dimer of two C-termini containing a CaM attached at each end [308]. Each CaM twists around three alpha-helices, whereas two are from one CaM-binding domain and one is from the other CaMBD subunit. These findings have suggested that a CaMBD dimerization process induced via the Ca^2+^/CaM complex establishes SK channel gating [308,309].

Structural resolutions of the SK4 channel in complex with CaM have suggested that the C-terminal CaM lobe (C-lobe) is constitutively bound to SK4, whereas the N-terminal lobe (N-lobe) controls SK4 gating in a Ca^2+^-dependent manner [304] (Figure 7). Indeed, in the Ca^2+^-free environment, the cryo EM structure reveals a tight association of the CaM C-lobe to the H_A_ and H_B_ helices, while the CaM N-lobe displays high mobility. The latter likely allows fast detection of and response to the local Ca^2+^ signals [310]. In the presence of Ca^2+^, the cryo EM structure reveals that the CaM N-lobe is attached to SK4, thus, forming a novel interaction network. Specifically, CaM binds to the S1 and S2 helices and directly contacts the H_A_ and H_C_ helices of a neighboring subunit. Overall, each CaM molecule has been reported to interact during SK channel activation with three subunits of the SK channel tetramer. The binding pocket for CaM is formed by S_45_A, with S_45_B forming a bridge to S6 which enables the indirect contact of CaM to the pore. In support, several residues of S_45_A helix face the CaM N-lobe pocket directly. This region of the helix is highly conserved among the SK channel family potentially reflecting its importance for the channel function. Moreover, several amino acids, that form interactions within one subunit and between two subunits (N201 with R287 and K197 with E295, respectively) have been reported to hold the structural elements together. The gate formed by the residues V282 at the S6 helices represents the narrowest part of the channel pore with a radius <1 Å in the closed state. The SK4 V282G mutant has been reported to form a leaky channel that allows activation of currents also in the absence of Ca^2+^. Moreover, two disease-related gain-of-function mutants SK4 V282Q and SK4 V282M associated with a type of hemolytic anemia are currently known [304].

Overall, structural and functional analysis have demonstrated that the C-lobe is responsible for Ca^2+^-independent tight association to the SK channel subunit, whereas Ca^2+^-induced gating is established via EF hands in the N-lobe. Ca^2+^-bound CaM triggers structural alterations within the SK channel that leads to pore opening [304,306]. The current idea of the SK channel activation mechanism suggests that upon the increase of [Ca^2+^]_i,_ CaM N-lobe binds to Ca^2+^ ions, which induces a conformational change. Hence, the affinity of the N-lobe for binding to S_45_A helix is enhanced. Subsequently, CaM couples to the S_45_A and moves it toward the cytosol, whereas S_45_B helix is displaced from the pore axis (Figure 7c). This movement leads to structural changes of the S6 helices allowing pore opening. Ca^2+^-independent CaM binding has been shown to control the SK channel trafficking to the membrane [311].

## 6. SK Channel Pharmacology

Diverse natural substances extracted from sea anemones, scorpions, or bee venoms have been reported to interfere with the function of SK channels. The most commonly used represents the neurotoxic peptide from the bee venom, apamin [312]. While it blocks SK channels efficiently without acting on BK and IK channels, it is non-selective among the SK isoforms. Since the discovery of apamin, an arsenal of peptides and small-molecule inhibitors that possess either positive or negative modulatory effects is currently available. Among the positive modulators, 1-EBIO and riluzole do not distinguish between the four SK channels [313,314,315], while NS309 activates SK1, SK3, and SK4, but not SK2 [316]. More selective ones are SKA-111 and SKA-121 for SK4 and CyPPA and NS13001 for SK2/3 [314]. Among the negative modulators dequalinium, NMAG525E1, NS8593, and ICAGEN inhibit SK1–3 channels, while RA-2, represents a non-selective SK channel blocker [314,315]. 4-AP and BMB are selective blockers for SK3 channels [317] (Table 3). While positive modulators shift the Ca^2+^ concentration-dependent activation of SK channels to lower Ca^2+^ concentrations, negative modulators cause a shift to higher Ca^2+^ concentrations [318]. For most of the activators, the working mechanism is unclear. Nevertheless, it has been reported that the binding pocket for 1-EBIO, CyPPA, NS309 and SK-111 is located at the interface between the S_45_A helix and the CaM N lobe, thus, potentially stabilizing the CaM-SK channel complex [319]. Interestingly, most of the SK4 inhibitors seem to couple to the same site in the inner of the pore. Apamin binds at the outer side of the pore complex at the S3-S4 extracellular loop and triggers its inhibitory effect allosterically [320]. The antibacterial drug dequalinium chloride and its synthetic analogues have all been reported to act at the apamin-binding site [320].

Only recently, the charged phospholipidic molecule edelfosine has been found to act as an inhibitor on SK3 via interfering with lipid rafts. Because of its toxic effect, a novel ether-lipid ohmline has been synthesized and appeared as a selective inhibitor of SK3 channels. Novel amphiphilic compounds composed of a tetrahydropyridine group similar to apamin and a saturated or unsaturated fatty chain only recently arose as selective inhibitors of SK channels [321]. Mechanistically, it has been reported that ohmline interferes with the order of lipid bilayers rich in cholesterol. Whether there is a direct interaction between ion channels and these ether lipids is currently unknown [322]. Nevertheless, it has recently been reported that SK3 possesses a PIP_2_-binding site making a specific interaction of SK3 and such lipid-synthetic alkaloids likely [39].

## 7. Ca^2+^-Activated K^+^ Channels in Diseases

### 7.1. SK Channel in Neurons and Neuronal Disease

SK channels play an essential role in neurons for the intrinsic excitability and synaptic function. Dysregulation of SK channels has been connected to neuropsychiatric/neurodegenerative disorders such as epilepsy, Parkinson’s disease, schizophrenia, or bipolar disorder [275,295]. In human patients diagnosed with schizophrenia, a spontaneous N-terminal deletion mutation of the SK channel gene has been detected [331,332]. Similarly, significantly suppressed expression and function of SK channels is responsible for the development of epilepsy [333]. Down-regulation of SK channels has been determined after induced status epilepticus (30 min continuous seizure or repeated seizures). The role of SK channels in Parkinson’s disease has remained elusive because of the contradictory evidence. Although some reports have demonstrated that enhanced SK channel activity could mitigate symptoms of Parkinson’s disease [270,334,335,336,337]. The reason for the different results is probably that Parkinson’s disease consists of different stages. Besides the role of the SK channels in neurons and epithelial cells, several publications have already highlighted their role in breast, colon, or prostate cancer cells.

Several SK3 gain-of-function mutations (K269G, G350N, S436C, V450L) (Figure 7a) have been associated with a rare developmental disorder, the Zimmermann–Laband Syndrome. As patients show, among diverse phenotypes, in addition epilepsy, this syndrome has been proposed to belong to neurological channelopathies [338].

### 7.2. SK Channels in Cancer

With respect to the potential role of the K_Ca_ channels in cancer only a few studies are currently available. Interestingly, SK channels have been reported to be expressed only in four cancer types. Gene expression of at least one of the K_Ca_ members has been identified in medulloblastoma (SK3) [339], glioma (SK2) [303], melanoma (SK2 and SK3) [297], or breast cancer (SK2 and SK3) [340]. Interestingly, despite the detection of gene expression in medulloblastoma and brain tumor cells, no SK-typical current activation or other biological effects have been detectable. Thus, diverse reports have assumed that the presence of the gene does not necessarily lead to the subsequent expression of the functional protein [297,341,342,343]. Contrarily, in breast cancer and melanoma cells, both SK2 and SK3 channel activity have been proven. Additionally, in colon cancer cells SK3 expression and SK3-mediated currents have been detectable. In particular, SK3 channel activity drives breast and colon cancer cell migration, which is abolished by the SK channel inhibitor apamin. In melanoma cells, SK3 channel expression controls cell motility. The proliferation enhancing the role of SK2 in melanoma cells appears only under hypoxia [269,343] (Table 2).

The main focus in this review is laid on the co-regulation of SK channels with the CRAC channel components. Nevertheless, among other members of the Ca^2+^-activated K^+^ channel family, both, IK and BK channels, have been found to play a significant role in cancer. The association of BK and IK channels to specific cancer hallmarks and tumor progression has already been reported for many diverse tumor cell lines such as breast, prostate, colon, glioblastoma, melanoma, cervical carcinoma, and others. This knowledge has already been discussed in many excellent reviews [344,345,346,347,348,349,350,351,352,353]. Specifically, BK channels have been found to determine glioma, breast, and prostate cancer cell growth [276,299,354,355,356,357,358] (Table 2). IK channels are involved in the control of the typical cancer hallmarks of glioma, colon, prostate, breast cancer, and cervical carcinoma [43,64,273,296,303,359,360] (Table 2). The most frequently occurring type of cancer-cell-hijacked biological function appears to be the upregulation of the protein expression which induces cell proliferation, migration, and finally triggers bone metastasis [344,345,346,347].

## 8. Individual and Collective Modulation of Ca^2+^ Sensitive Ion Channels in Lipid Rafts

There is rapidly accumulating evidence that the role of ion channels in cellular signaling processes is not only determined by the pore-forming complex by itself but also depends on the molecular components in their environment, the so-called micro- and nanodomains. Such membrane areas are known as lipid rafts. They are enriched with cholesterol and sphingolipids, which facilitate the assembly of a series of signaling molecules and serve as sorting platforms for signal transduction molecules. Here, ion channel function is modulated by proteins and lipids that either directly interact with or allosterically influence the respective ion channel. Hereby, a precisely controlled sequence of cellular signaling events, including for instance exocytosis and endocytosis, cell adhesion and migration, organization of the cytoskeleton and apoptosis, are guaranteed [300,361,362].

In contrast to non-tumorigenic tissue, cancer cells include higher amounts of cholesterol and lipid rafts. A multitude of events triggering cancer cell development and progression depends on lipid rafts and their modulation. Besides the pharmacological interference with diverse signaling steps in the ion channel activation cascade, pharmacological lipid raft modulation represents a promising strategy to interfere with the progression of cancerous diseases [361,362].

**Figure 8:**
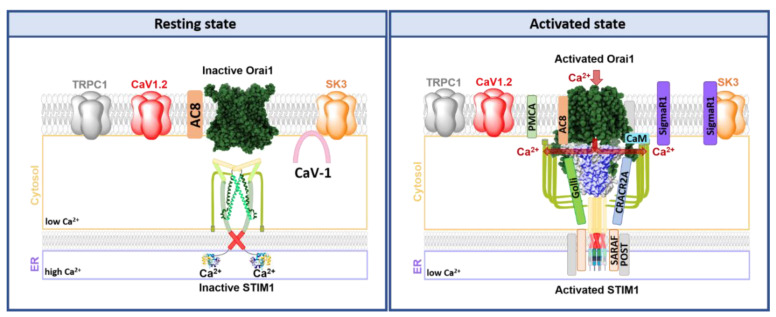
Resting and activated state of SOCE consisting of Ca^2+^ related ion channels in close proximity to or in complex with diverse accessory proteins. Under resting conditions with high Ca^2+^ concentrations in the ER, STIM1 and Orai1 remain in the quiescent state. In dependence of the cell type located in close proximity to TRPC1, CaV1.2, or interplay with adenylyl cyclase 8 (AC8) and/or SK3. Caveolin-1, proposed to interact with Orai1 as well as SK3 channel, is depicted in the inner face of the plasma membrane (**left panel**). Upon Ca^2+^ store depletion, CRAC channel complex formation is modulated by accessory modulators such as Golli, CRACR2A, Calmodulin, SARAF, and POST (**right panel**). Interaction of adenylyl cyclase to activated Orai1 triggers downstream signaling processes such as cAMP-PKA pathway [363] (not shown). CRAC channel complexes can form within caveolae. There, STIM1 potentially interacts with and activates TRPC1. Interaction of SK3 channel with accessory modulator SigmaR1 is known to support SK3 and Orai1 co-localization to promote Orai1-dependent SK3 channel activation as outlined in Section 9.

In the following, we will provide an overview of the currently known regulatory roles of lipids and proteins (Figure 8) on CRAC as well as Ca^2+^-activated K^+^ channels.

### 8.1. CRAC Channel Regulation in Signalplexes in Lipid Rafts

#### 8.1.1. Lipid Mediated STIM1-Orai1 Regulation

The most prominent lipids that function as essential regulators of a diversity of ion channels represent PIP_2_ and cholesterol [364,365,366,367].

It is increasingly clear that lipid rafts define the assembly and regulation of the STIM1/Orai1 machinery with PIP_2_ and cholesterol playing the most important role. The phospholipid PIP_2_ located in the inner leaflet of the cell membrane has been demonstrated to regulate STIM proteins. The C-terminal end of both, STIM1 and STIM2, contains a lysine-rich region, which functions as a PIP_2_-binding site [105,368]. Sufficient PIP_2_ and PIP_3_ levels in the plasma membrane, which are especially enriched in lipid rafts, promote a stable coupling of STIM to the plasma membrane, its targeting to ER-PM junctions and the clustering of STIM1 and Orai1 [368,369,370,371]. PIP_2_ levels in the plasma membrane are regulated by several proteins, which have been also shown to influence STIM1-Orai1 activation. These include septins, E-Syt1, Nir2, and RASSF4 [372,373,374,375,376,377]. Septins represent GTP-binding proteins in the plasma membrane that bind PIP_2_. They have been reported to organize ER-PM junctions, which promote STIM1-Orai1 clustering thereby enhancing SOCE activation [372]. E-Syt1 functions as an ER-to-PM tether and recruits Nir2 to ER–PM junctions where it mediates PIP_2_ replenishment after cell stimulation. Both E-Syt1 and Nir2 are co-localized with STIM1 within these junctions to prime the cell for additional stimulation. A regulatory protein RASSF4 has been shown to control the steady-state PIP_2_ levels in the plasma membrane, thus, controlling ER-PM junctions and the activation of STIM1-Orai1 [374,375].

Cholesterol, a hydrophobic lipid, located within the hydrophobic layer of the membrane [378], has been reported to modulate the function of a diversity of ion channels (nAChR, Kir, BK, TRPV [379]). Moreover, STIM1 and Orai1 have been discovered to bind cholesterol via the so-called cholesterol recognition amino acid consensus motif (-L/V-(X)_(1-5)_-Y-(X)_(1-5)_-R/K-). Their interaction interferes with STIM1-mediated Orai1 activation in an inhibitory manner [380,381,382]. Cholesterol depletion has been found to enhance Ca^2+^ currents in STIM1/Orai1 expressing HEK cells. Moreover, endogenous CRAC currents in mast cells together with mast cell degranulation has been discovered to be enhanced [380]. The latter has been associated with the Smith-Lemli-Opitz syndrome a disease where patients suffer from hypocholesterolemia as well as enhanced allergy response [383].

#### 8.1.2. Proteins Modulating STIM1-Orai1 Function

STIM1 and Orai1 are sufficient to constitute the CRAC channel. Nevertheless, emerging insights into the STIM1/Orai1 regulation reveal an array of regulatory proteins, which are involved in the modulation of STIM1 and/or Orai1 [104,254,369,370,371,376,384,385,386,387,388,389,390,391,392,393,394,395,396]. This allows the CRAC channel components to trigger a wide range of signaling events and adapt to the regulation of cells in disease. They act via direct binding to cytosolic STIM1 or Orai1 segments either in a positive or negative regulatory manner on the CRAC channel signaling machinery as described in the following (Figure 8).

The quiescent and active state of STIM1 is not only controlled by the Ca^2+^ levels in the stores, but additionally by the accessory proteins. While SARAF and SigmaR1 negatively modulate STIM1/Orai1 activation, STIMATE acts as a positive modulator [394,397,398,399,400,401]. In addition, several other proteins are involved in modulating STIM1 function, as described in more detail in the following paragraphs (Figure 8).

SOCE-associated regulatory factor (SARAF) is a single-pass TM protein located in the ER. It keeps STIM1 in an inactive state via coupling to the STIM1 C-terminal region close to the C-terminal inhibitory domain (CTID, aa: 448–539). Upon store-depletion SARAF dissociates from STIM1, to allow activation of Orai1. Subsequently, SARAF couples again to STIM1, which has been reported to facilitate SCDI [399]. Moreover, upon store-depletion an interaction of SARAF with Orai1 C-terminus has been determined. Their interplay enhances Ca^2+^ entry via Orai1 independent of STIM1 [397]. SARAF has been further elucidated to control PM localization of STIM1 [398].

STIM-activating enhancer (STIMATE), a multi-transmembrane, ER-located protein, promotes clustering of STIM1 and subsequently CRAC channel activation. Silencing of STIMATE significantly reduced store-operated Ca^2+^ entry. STIMATE has been reported to tightly co-localize and interact with STIM1, but not with Orai1. Mechanistically, STIMATE couples to STIM1 CC1 thereby interfering with the inhibitory clamp of STIM1, promoting STIM1 clustering and subsequent store-operated Ca^2+^ entry [400].

SigmaR1, a stress-activated chaperone protein, has been demonstrated to reduce store-operated Ca^2+^ entry. It binds to STIM1 and upon store-depletion, it slows its recruitment to Orai1 in the plasma membrane [388].

POST is a 10-transmembrane protein located both in the ER and the plasma membrane. While it binds to Orai1 in a store-independent manner, it couples to STIM1 upon store-depletion. It does not affect store-operated Ca^2+^ entry, however, decreases Ca^2+^ ATPase activity (SERCA, PMCA) in the plasma membrane. This leads to reduced levels of Ca^2+^ store repletion and thus, enhances NFAT activity [396].

Junctate, a Ca^2+^-sensing protein, forms a structural component of the ER-PM junctions. An EF-hand mutant junctate has been suggested to facilitate the clustering of STIM1 at the ER-PM junctions and enhances the probability for STIM1-Orai1 coupling [402].

CaM has been reported to interplay with STIM1, to dissociate the STIM1-Orai1 complex and lead to deactivation. The CaM-binding site is located close to the STIM1-Orai1-binding site within STM1 [403].

The most important proteins modulating Orai1 activity are described in the following:

The Ca^2+^-release-activated channel regulator (CRACR2A) represents a Ca^2+^ sensor in the cytosol containing two EF-hand domains. It has been discovered to cluster with Orai1 and STIM1 via direct interaction, thus forming a ternary complex. CRACR2A has been shown to enhance STIM1 mediated Ca^2+^ entry via Orai1. Their co-regulation occurs in a Ca^2+^-dependent manner. At high cytosolic Ca^2+^ concentrations CRACR2A dissociates from STIM1 and Orai1, while at low Ca^2+^ levels their association is enhanced, thereby promoting SOCE. The CRACR2A-binding site in Orai1 is located in the N-terminal region between the amino acids 64 and 93. Site-directed mutagenesis of two positively charged residues K85 and K87 to alanine has abolished the coupling of CRACR2A with Orai1 and subsequent clustering with the STIM1-Orai1 complex. The CRACR2A-binding site within STIM1 represents the CC region and proline/lysine-rich region, but is not located within the STIM1 SOAR domain [393]. CRACR2A in co-regulation with STIM1 and Orai1 has been recently suggested to potentially act as an upstream regulator of prostate cancer progression [404] (Figure 8).

Golli proteins, isoforms of myelin basic proteins, have been shown to co-localize with Orai1 upon store-depletion. This led to the assumption that Golli competes with Orai1 in coupling with STIM1. Moreover, Golli has been reported to trigger the Ca^2+^-dependent inactivation of CRAC channels. The negative modulatory effect of Golli is assumed to be established via its coupling to STIM1 C-terminus [386]. There is evidence that Golli localizes via its myristoylation site on its N-terminus in lipid rafts [382].

Caveolin-1 has been reported to reduce SOCE during meiosis, potentially because of the internalization of Orai1 and impairment of STIM1 clustering. Orai1 trafficking seems to be triggered via an N-terminal-binding site within Orai1 [9,10,381] (Figure 8).

Ca^2+^-sensitive adenylyl cyclase 8 (AC8) is constitutively bound to Orai1 N-terminus and generates cyclic adenosine monophosphate (cAMP), which subsequently activates protein kinase A (PKA) [405]. The latter phosphorylates S34 in the N-terminus of Orai1 to trigger CDI. In contrast, the recruitment of the phosphatase calcineurin reverses the effect of the PKA [363] (Figure 8).

Additionally, phosphorylation sites, both in STIM1 as well as Orai1, possess a regulatory role in CRAC channel activation. In particular, Orai1 N-terminus includes two serines, S27 and S30, which increase the activation of CRAC channels upon mutation. This proposes that protein kinase C (PKC) impairs CRAC channel activation upon phosphorylation of these sites [406]. The residue Y361 in CC2 is phosphorylated by the proline-rich kinase 2 upon store-depletion. Knocking out this phosphorylation site (Y361F) still leads to STIM1 punctae formation, but coupling to Orai is completely abolished [407]. Another STIM1 phosphorylation site, Y316, has been reported to modulate the interplay with SARAF and CRAC channel activation [408]. Furthermore, this mutation has been identified to modulate the interaction with SARAF [408]. STIM1 C-terminus comprises the phosphorylation sites: S468 and S668 which modulate store-depletion-induced Ca^2+^ currents during meiosis and mitosis [409,410,411]. Additionally, STIM1 is modulated by extracellular-signal-regulated kinases 1 and 2 (ERK1/2) [412], which lead to the phosphorylation of certain sites within the STIM1 C-terminal serine/proline-rich region.

#### 8.1.3. CRAC Channel Components in Co-Regulation with Other Ion Channels

In addition to lipids and proteins mentioned above, several studies have reported an interplay of one of the CRAC channel components with other ion channels, including TRP channels, SPCA2, and Ca^2+^-activated ion channels. Their co-regulation typically involves rapid and specific rearrangement of critical proteins within the cell that are responsible for Ca^2+^ entry and relaying intracellular Ca^2+^ signals.

Induction of store-depletion has been shown to transiently enhance the co-localization of STIM1 with Orai1, TRPC1, and TRPC6. Lipid raft disruption has been shown to attenuate or abolish their association [395]. Overall, it has been elucidated that rather the activation than the maintenance of store-operated currents requires the presence of lipid rafts. Moreover, STIM1-induced Orai1 currents have been shown to trigger the trafficking of TRPC1 channels to ER-PM junctions, where they are activated by STIM1. The co-regulation of STIM1 and TRPC1 is further supported by Caveolin-1, which directly interacts with TRPC1. Disruption of lipid rafts reduced STIM1 puncta formation as well as the co-localization with TRPC1 (Figure 8). Besides the occurrence of STIM1 in the ER membrane, it has been detected in the plasma membrane. The latter requires to be localized in lipid rafts to exert an inhibitory effect on STIM1-mediated Orai1 currents [413]. Reports on human platelets suggest that lipid rafts recruit TRPC1, TRPC4, and TRPC5 in signalplexes [414,415] which facilitates the assembly between TRPC1, STIM1, and Orai1 [389]. Moreover, in vascular and coronary artery smooth muscle cells, TRPC1 and Orai1 have been found to co-localize with the voltage-gated Ca^2+^ ion channel, CaV1.2 [416,417] (Figure 8).

The secretory pathway Ca^2+^-ATPase (SPCA2), has been elucidated to induce constitutive Ca^2+^ entry via Orai1 in a breast cancer cell line. It binds directly to the N- and C-terminus of Orai1 independent of STIM1 and store-depletion (aa 48–91) [254]. Obviously, gating of Orai1 can be obtained via a distinct mechanism than that via STIM1. Enhanced Ca^2+^ levels triggered by the SPCA2–Orai1 complex activate the Ras–ERK pathway, which is involved in proliferation and cell cycle and a relevant number of human cancers [255]. The upregulation of SPCA2 has been supposed to come along with certain malignancies such as colon or breast cancer. Moreover, SPCA2 has been reported to promote microcalcification in certain carcinomas, as a phenomenon linked to more aggressive forms of cancerous diseases. The knockdown of SPCA2 has been reported to attenuate proliferation and tumorigenesis [254,418].

Orai channels have been further described to interplay with *K^+^ ion channels*. Especially, in breast cancer cells it is been found that Orai1 interplays with the Ca^2+^-activated K^+^ ion channels, SK3, and the voltage-dependent Kv10.1 ion channel. Both have been reported to promote Ca^2+^ entry via Orai1 [255].

In Section 9 of this review, we aim to focus especially on the so far best-studied co-regulation of Ca^2+^-activated K^+^ ion channels (K_Ca2+_) with CRAC channel components. Prior to this, we describe in the following the current knowledge on the regulation of K_Ca2+_ ion channels in micro-/nano-domains.

### 8.2. Ca^2+^-Activated K^+^ Channel Regulation in Microdomains

#### 8.2.1. Modulation of K_Ca2+_ Channels by Lipids

CaM-dependent regulation of SK channels is additionally modulated by PIP_2_, as depletion of PIP_2_ leads to SK2 channel inhibition. The PIP_2_-binding site is located at the boundary layer of the SK-CaM-binding sites. It has been shown that phosphorylation of CaM reduces the affinity of SK2 to PIP_2_, because of the altered interactions of amino acids contributing to the PIP_2_-binding site [419].

There is clear evidence for cholesterol-mediated regulation of all types of Ca^2+^-activated K^+^ channels. Cholesterol-mediated regulation of SK channels occurs in dependence of caveolin-1, while BK and IK channel function is modulated by cholesterol independent of caveolin. Cholesterol has been reported to possess an inhibitory effect on BK channels, likely because of a change in the open probability, but not the unitary conductance. Despite these facts, the detailed molecular mechanisms of cholesterol-mediated K_Ca2+_ channel modulation remain unclear [366].

#### 8.2.2. Accessory Proteins Modulating K_Ca2+_ Channel Activity

Besides CaM, which represents the most prominent molecular interaction partner of SK channels, as outlined in detail in previous sections, there are a few other associated regulatory proteins known to impact the SK channel activity.

The latter includes the protein kinase CK2 and the protein phosphatase 2A. They function especially as regulatory components of SK2 and SK3, and co-assemble together with CaM at the CaMBD and the N-terminus of SK2/3. The protein kinase additionally interacts with the C-terminus of SK channels. CaM, specifically T80, represents a target for phosphorylation by the protein kinase CK2 critical for SK channel function. Dephosphorylation decreases the affinity of Ca^2+^ to CaM and shifts the sensitivity of SK channels to Ca^2+^ to the submicromolar range. This protein complex guarantees that SK channels can adapt to physiological functions in a cell-type specific manner [300].

In cardiomyocytes, the cytoskeletal protein α-actinin 2 has been reported to interact via its EF hand domain with the SK2 channel [420] specifically the CaM-binding domain [421].

BK channels, sensitive to Ca^2+^ in the micromolar range, form stable macromolecular complexes with a set of BK -ß and γ subunits that fine-tune Ca^2+^ sensitivity, voltage-dependence and ensure the range of diverse functional features in cell signaling [422,423].

#### 8.2.3. K_Ca2+_ Channel Activity in Co-Regulation with Other Ion Channels

The activation mechanism of SK channels indicates that they might be co-localized with Ca^2+^ ion channels. Indeed, both, voltage-gated as well as non-voltage-gated Ca^2+^ ion channels form important Ca^2+^ sources allowing the activation of diverse K_Ca2+_ channels, which in turn enhance the membrane potential. In excitable cells, K_Ca_ channel-mediated hyper- or repolarization closes voltage-gated ion channels and thus, reduces Ca^2+^ entry via a negative feedback mechanism. In non-excitable cells, the activation of K_Ca2+_ channels enforces the driving force for Ca^2+^ to enter the cell [305] because of a positive feedback mechanism. An interplay of Ca^2+^ and K_Ca2+_ channels has been elucidated for the following ones.

Several reports indicate that different Ca^2+^-dependent K^+^ and voltage-dependent Ca^2+^ channels colocalize and couple to each other. For instance, the functional coupling has been observed between voltage-dependent Ca^2+^ and BK channels in diverse cell types, such as L-type and N-type channels in the active zones of hair cells [424,425] as well as neocortical pyramidal neurons [426] or L- and Q-type channels in adrenal chromaffin cells [427]. Additionally in dopaminergic neurons, T-type Ca^2+^ channels have been demonstrated to be coupled to small conductance SK channels [428]. BK channels and L-type Cav1.2 channels have been shown to co-localize and interact in rat brain and adrenal chromaffin cells [301,428,429]. Single-channel experiments have revealed that L-type Ca^2+^ channels are specifically coupled to SK channels [430].

The assembly of BK channels with high voltage gated Ca^2+^ ion channels Cav1.2, Cav2.1, Cav2.2 as well as Cav3.2 has been characterized in more detail. Interaction of these different types of channels, partially verified via co-immunoprecipitation experiments, has suggested an interplay within nanodomains. For instance, K^+^ channels have been shown to colocalize and co-immunoprecipitate with Ca_V_3.2 in prostate cancer cells [299]. Direct interaction has been identified via the alpha-subunit of the Cav3.2 channels and the N-terminal TM domain of K_ca_1.1 [431]. However, a sufficient amount of Ca^2+^ ion channels, specifically their concerted activation, is required to allow robust activation of K_Ca_1.1 channel. This suggests rather an interplay at the microdomain level, despite their physical association. Thus, Ca^2+^ carried by one of these channel types is able to fuel co-assembled BK channels and subsequently K^+^ currents can be activated [432]. The interplay of BK and Cav3.2 channels plays an essential role in driving prostate cancer cell development [299].

Caveolin-1 has been reported to facilitate the interplay of BK and Cav1.2 channels [433].

BK channels are linked to IP_3_ receptors via lipid raft domains. It has been shown in glioma cells that the disruption of lipid rafts abolishes the interplay of these molecular components [434].

Besides the voltage-gated Ca^2+^ ion channels, diverse non-voltage-gated Ca^2+^ ion channels have been shown to interplay with K_Ca2+_ channels. Orai1 has been detected to co‑immunoprecipitate and colocalize with SK4 upon overexpression in HEK 293 cells [72]. In human lung mast cells, SK4 has been suggested to be highly dependent on Orai1 mediated Ca^2+^ influx via a close spatiotemporal interaction [72]. Selective inhibition of Orai1 or the expression of the Orai1 E106Q pore mutant has deceased SK4 channel currents. Co-immunoprecipitation experiments have revealed only an interaction of SK4 with Orai1 but not with Orai2 [72]. In microglial cells, SK4 and Orai1 have been detected in close proximity, which controls microglial migration [435].

Additionally, there is evidence that an interplay of K_Ca_ and non-voltage gated Ca^2+^ channels can determine cancer cell growth [36,43,271,340]. SK4 channels have been demonstrated to co-immunoprecipitate with TRPV6 in LNCaP cells [296]. Further, Kv10.1 has been reported to associate with Orai1 and to control breast cancer cell migration through Orai1-dependent Ca^2+^ entry [436].

**Figure 9:**
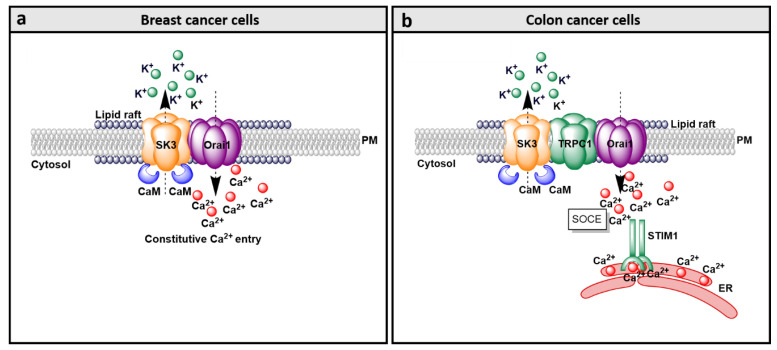
SK3 and Orai1 interplay in different cancer types. (**a**) Colocalized SK3 and Orai1 within lipid rafts exhibit constitutive Orai1-dependent Ca^2+^ entry independently of STIM1 in breast cancer cells. (**b**) SK3/TRPC1/Orai1 complex triggers SOCE-dependent cancer cell migration in colon cancer cells.

Moreover, close proximity of SK and BK channels with Orai1 has been reported [437,438]. Specifically, a complementary approach of biochemical, fluorescence microscopy, and electrophysiological approaches has consistently suggested that Orai1 BK channels associate in rat mesenteric artery smooth muscle as well as in HEK293T cells [437,438]. Additionally, SK3 and Orai1 in breast cancer cells have been determined via immuno-colocalization [38,340]. In colon cancer cells, the recruitment of Orai1-TRPC1 channels into lipid rafts containing SK3 channels is further triggered by STIM1 [272] (Figure 8 and Figure 9). Thus, the physical association of SK3 and Orai1 is likely, however, the experimental proof is still lacking [271]. The current knowledge on the SK3-Orai1 interplay is covered in detail in the following section.

## 9. SK3 and Orai1 Channel Interplay

Currently, the co-regulation of SK3 and Orai1 represents one of the best-studied examples for an interplay of two types of Ca^2+^-regulated ion channels in cancer. In the following, we provide a detailed overview of the current knowledge on the SK3-Orai1 interplay and accentuate the still unresolved questions.

### 9.1. SK3 and Orai1 Colocalization in Lipid Rafts in Cancer Cells

Healthy human breast, prostate, and colon cells express only Orai1. Contrarily, related cancer cells exhibit a co-expression of Orai1 and SK3 channels. These two types of ion channels were identified to co-localize and interplay in lipid nanodomains [34,44,72,271,272,435], where they govern cell proliferation, migration, or bone metastasis [34,38,271,272,298,439].

In breast cancer cells, the interplay of SK3 and Orai1 has been demonstrated to lead to constitutive Orai-dependent, but STIM1-independent Ca^2+^ entry. In support, only knock-out (KO) cells of Orai1, but not of STIM1 have been shown to impair cell migration [34]. The co-localization of SK3 and Orai1 in lipid raft domains reinforces cancer cell growth. Disruption of lipid rafts by alkyl ether lipids edelfosine and ohmline has interfered with SK3-Orai1 colocalization and resulted in the loss of constitutive Ca^2+^ entry, cell migration, and bone metastasis [34,38,271,272,298,439] (Figure 9a).

In colon cancer cells, SK3 has been reported to colocalize not only with Orai1, but also with TRPC1 channels in lipid rafts. Their co-regulation involves additionally STIM1 and is strengthened via three positive feedback loops. First, the recruitment of TRPC1/Orai1 complexes into lipid rafts is mediated by phosphorylated STIM1 upon store depletion [272]. Phosphorylation of STIM1 occurs by EGF and stimulation of the PI3K/Akt pathway. Second, the activation of SOCE at SK3/Orai1/TRPC1 complexes increases Akt which enhances phosphorylation of STIM1 and in turn promotes SOCE. Third, phosphorylated Akt (P-Akt) stimulates a small protein Rac1, which increases SOCE and in consequence P-Akt. These three loops contribute to the amplification of SOCE and SK3-dependent migration. The localization of the SK3/Orai1/TRPC1 complex in lipid rafts is supposed to be supported by direct interaction with Caveolin-1 with Orai1 and TRPC1. The SK3/Orai1/TRPC1 interplay is proposed to interplay with the SK3 channel to trigger SOCE-dependent cancer cell migration. Furthermore, the enhanced activity of Ca^2+^-dependent protease calpain has been described to contribute to increased cancer cell migration. In line with the observations in breast cancer cells [34], in colon cancer cells, SK3 moves apart from the complex outside of the lipid rafts upon the application of ohmline [272] (Figure 9b).

Overall, these data propose that the formation of SK3-Orai1 complexes in lipid rafts is crucial for cancer cell development [305]. Impairment of SK3-Orai1 co-localization alters the cellular machinery and suppresses cancer cell migration. Edelfosine and ohmline derivates, which act inhibitory on the SK3-Orai1 interplay, either because of a direct effect on the ion channel function or rearrangements of lipid rafts, exhibit promising anti-cancer properties. However, because of the side effects, they have been excluded from clinical therapeutic trials.

Despite solid evidence that the SK3/Orai1 interplay plays a significant role in cancer cell growth, several questions remain to be answered. How is the tight co-localization of SK3 and Orai1 stabilized? Which structural determinants, both, within SK3 and Orai1 mediate their interplay in lipid rafts? How is STIM1 affecting the SK3-Orai1 co-regulation? Especially, within the CRAC channel field, an arsenal of mutants is available that could help to unravel crucial sites that establish the interplay of Orai1 and SK3 channels [66]. It might be expected that the well-known Orai1 E106Q pore mutants or several other loss-of-function mutants (e.g., Orai1 K85E) abolish the interplay with the SK3 channels. It is likely that the cytosolic segments of Orai1 interplay with SK3, which might be investigated via the well-known N- and C-terminal Orai1 deletion and single point mutants. Among the diverse Orai1 mutants, a bunch of gain-of-function mutants are currently known, which might be investigated for a potential amplification of the Orai1-SK3 co-regulation. Furthermore, a series of STIM1 mutants interfering with one of the diverse STIM1 activation steps are available, which represent highly valuable tools to determine how STIM1 impacts the SK3-Orai1 interplay.

Up to date, the formation of the BK- or IK – Orai1 channel complexes and their potential role in cancer cell function has not yet been clarified in detail. Nevertheless, given the similarity of calcium-dependent activation among the K_Ca_ family members, it is tempting to speculate a plausible association of such complexes to proliferation and migration of cancer cells. Initial attempts focusing on such channel complexes already provided a hint that Orai1 is able to form a signal complex with BK channels in mesenteric artery smooth muscle cells [437] or physically associate to BK channels and promote their activity in HEK293T cells [438]. Summarized, BK/IK/SK–Orai1 channel complexes represent new potentially highly attractive candidates to govern cancer cell fate and develop new therapeutic approaches.

### 9.2. SK3/Orai1 Complex and Accessory Proteins

Currently, it is unknown whether the interplay of SK3 and Orai1 in lipid rafts is established via a direct interaction. It is worth noting that BK channels have been found to get in close proximity to Cav2.3 channels via a direct interaction [299], as described above. Such an interplay might be conceivable for SK3 and Orai1, but still requires further investigations. Alternatively, their SK3-Orai1 complex formation might be facilitated by accessory proteins.

**Figure 10:**
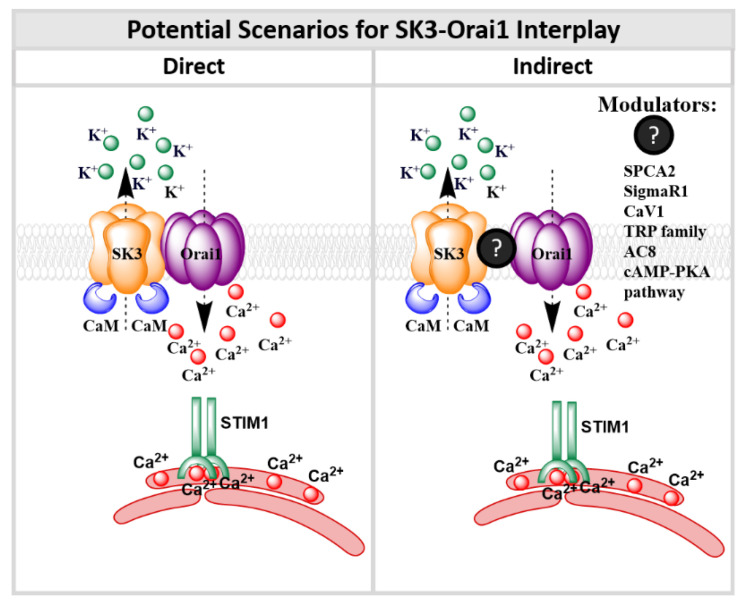
Potential scenarios of SK3-Orai1 interplay. The direct interaction and tight co-regulation between SK3 and Orai1 and potentially STIM1 (**right panel**). The indirect interaction between SK3- and Orai1-mediated through probable modulators such as SPCA2, SigmaR1, Caveolin-1, TRP family or members of cAMP-PKA pathway (**left panel**) in addition to the possible role of STIM1.

There is profound evidence that Ca^2+^ ion channels exist in complexes with other functional proteins [440]. As mentioned above, despite STIM1 and Orai1 are sufficient to fully reconstitute the CRAC channel [441], a series of regulatory proteins have meanwhile been identified that regulate their interplay [384]. These include, for example, a stress-activated chaperone, SigmaR1, and the Ca^2+^-ATPase, SPCA2 [32,271,340] (Figure 10).

Interestingly, with respect to the co-regulation of SK3 and Orai1, there is solid evidence that SigmaR1 interplays with STIM1 [388] as well as SK3 [271]. While SigmaR1 and STIM1 interact directly in HEK 293 cells [388], in breast and colon cancer cells, this receptor associates directly with SK3 and triggers a close association of Orai1 and SK3 [271]. It has been proposed that the reason for the different regulations in HEK293 and breast cancer cells may be that HEK 293 cells do not express SK3. Thus, in HEK293 cells SigmaR1 has been suggested to regulate CRAC channels via STIM1, thereby attenuating STIM1-Orai1 coupling [271]. It remains so far elusive whether a co-expression of STIM1, Orai1 and SK3 in HEK 293 cells or overexpression of STIM1 in breast cancer cells alters the interplay of the Orai1/SK3 channel complex.

Moreover, SigmaR1 has been reported to promote the interplay of SK3 and Orai1. In support, downregulation of SigmaR1 or the application of an inhibitory SigmaR1 ligand igmesine in breast and colon cancer cells has abolished the interplay of SK3 with Orai1 channels in lipid rafts and decreased their levels in lipid nanodomains [271]. Whether the direct interaction of SigmaR1 and SK3 promotes the co-localization or even interaction with Orai1 to facilitate their co-regulation remains still elusive. Hence, it remains to be investigated whether SigmaR1 interacts with Orai1. Interestingly, SigmaR1 has been reported to interact with diverse other ion channels and G-protein-coupled receptors. A direct physical association has been for instance reported for Kv1.2 α-subunit [442] and HERG with SigmaR1 [443], whereas the location of the binding sites has remained unclear. Moreover, it has been detected in a variety of cancer cells, whereas its role there is still weakly understood [444,445,446].

Interestingly, in analogy to SK3, SPCA2 has been reported to induce constitutive Ca^2+^ entry via Orai1 in breast cancer cells in a STIM1-independent manner. Obviously, SPCA2 and SK3 possess analogue roles in the regulation of Orai1 in cancer cells [254,447,448]. At this point, one key question is which mechanisms the co-regulation of SPCA2 and SK3 with Orai1 underlie. Moreover, as both, the SK3-Orai1 and SPCA2-Orai1 co-regulation have been elucidated in breast cancer cells, it is of interest whether SPCA2 and SK3 synergistically contribute to a co-regulation with Orai1 in lipid rafts in cancer cells and supports the initiation of constitutive Ca^2+^ entry via Orai1.

Besides the potential stabilizing role of accessory proteins on the SK3-Orai1 interplay, there is solid evidence that lipids facilitate their co-regulations. This assumption is based on the effects of alkyl-ether lipids disrupting the SK3-Orai1 co-localization and moving Orai1 outside of lipid rafts, thus, reducing cancer cell migration. These alkyl-ether lipids possess amphiphilic properties and integrate into the plasma membrane, where they are able to rearrange the lipid rafts, thereby interfering with the progress of cancerous diseases. The reorganization of lipid rafts by edelfosine has been demonstrated to induce the formation of CASMER, a cluster of apoptotic signaling molecule-enriched rafts, that initiate the apoptosis of the tumor. Additionally or alternatively alkyl-ether lipids are assumed to affect the function of ion channels and receptors responsible for apoptosis [39,317]. Ohmline and edelfosine showed high specificity for SK3 over other SK isoforms as well as other types of ion channels. However, the site of action of these alkyl-ether lipids is still unknown [39] and further studies are still required.

Overall, there is evidence that SK3 and/or Orai1 are modulated via accessory proteins as well as lipids, either when heterologously expressed in HEK 293 cells or when endogenously occurring in cancer cells. The detailed mechanisms of the modulatory proteins and lipids on either Orai1, SK3 or even both still represent an important question to be resolved in the future.

### 9.3. SK3/Orai1 Complex and cAMP-PKA Pathway

In addition to regulatory proteins and lipids, the cAMP-PKA pathway plays a role in the SK3/Orai1 co-regulation. Breast cancer cell migration is modulated by cAMP [449,450,451] via acting on the SK3-Orai1 interplay. Indeed, cAMP-enhancing drugs: isoprenaline (beta-adrenergic receptor agonist) or forskolin, (AC8 activator), have been found to reduce the basal Ca^2+^ levels, SK3 channel activity, and SK3-Orai1 complex formation in breast cancer cells. In consequence cancer cell migration is reduced [36]. In support, forskolin treatment has been demonstrated to displace the Orai1 channel outside of the lipid raft nanodomains, whereas SK3 remained. Moreover, the PKA inhibitor, KT 5720, recovered the effect of forskolin by 50%, indicating that SK3 channels are modulated by PKA phosphorylation. Overall, cAMP-elevating drugs are promising for therapeutic treatments of cancer cells.

## 10. Conclusions and Perspectives

In this review, we have shown that a deep knowledge of the structure-function relationship of CRAC and SK channels has been gained so far. In addition, there are indications of their co-regulation and their interaction with a large number of other molecular components within the cell. Currently, clear evidence is emerging that an interplay of Ca^2+^ and K_Ca2+_ ion channels is decisive in particular in the progression of cancer cell development and growth, as their downregulation reduces cancer cell proliferation and migration. Specifically, the interplay of Orai1 and SK3 has so far been intensively addressed and seems to be essential for breast and colon cancer cell growth. Nevertheless, the key molecular determinants mediating their interplay in signalplexes are still unknown. Their elucidation would be mandatory to develop novel, more specific therapeutic strategies against cancer. Within the past decade of intensive characterization of the key components of the CRAC channel machinery, valuable information on their activation mechanisms has been obtained. Moreover, a big toolbox of critical STIM1 and Orai1 mutants is available [48,66]. The latter are of particular importance in the continuing characterization of the key structures mediating the interplay of Orai1 and SK3 channels. Here, the prominent Orai1 pore mutant E106Q, diverse Orai1 truncation mutants, LoF and GoF mutants, as well as Orai1 dominant negative fragments [66], represent ideal candidates to narrow down key sites mediating the interplay with SK3 in signalplexes. For that, either an over-expression cell system or knock-in using the CRISPR/Cas9 technology in the particular cancer cells represent suitable strategies. Besides Orai1, the Orai family consists of Orai2 and Orai3. Specifically, Orai3 has been reported to play a role in breast cancer [59,258,285]. In breast cancer cells it has been reported that the SK3-Orai1 interplay occurs independently of STIM1 [34], while in colon cancer cells STIM1 contributes to the co-regulation of these two Ca^2+^ sensitive ion channels [272]. The reason for that and the diverse roles of STIM1 with respect to SK3-Orai1 complexes in cancer cells still requires further clarification. Thus, it remains of interest whether these isoforms somehow impact the SK3-Orai1 interplay. It is currently unknown whether the interplay of Orai1 and SK3 is triggered by direct or indirect interaction, whereas the latter seems to be more likely. Concerning Orai1, a series of regulatory proteins is known, such as SigmaR1 or SPCA2 [254,271], which might function as a link for the interplay of Orai1 with SK channels. The regulatory role of cholesterol on Orai1 channels is in line with the finding that breast cancer growth is determined by the co-localization of Orai1 and SK3 in cholesterol-rich regions. Whether STIM1 or Orai1 mutants deficient in cholesterol binding [380,382,413] impair the co-localization of the two types of ion channels remains to be investigated. Moreover, a set of pharmacological tools is available to interfere with and better understand the co-regulation of these ion channels.

The identification of key sites mediating Orai-SK interactions could provide novel target sites to interfere with cancer cell development. Our current detailed understanding of both types of Ca^2+^ sensitive ion channels serves as a fundamental basis for the detailed understanding of the molecular mechanisms of their coregulation as well as the development of more specific therapeutic strategies to interfere with cancer.

## Figures and Tables

**Table 1:** CRAC channel modulators. This table summarizes the most common CRAC channel modulators together with their empirical formula, the function of interest and some additional information.

Name	Empirical Formula	Function of Interest	Additional Information	References
2-APB	(C_6_H_5_)_2_BOCH_2_CH_2_NH_2_	Modulates: SOCE function in a concentration dependent manner	Activates: CRAC/STIM1 + Orai1 < 10 µM and Orai3 Blocks: CRAC/STIM1 + Orai1 > 50 µM Modulates: TRPV and TRPM channels	[206]
BTP2	C_15_H_9_F_6_N_5_OS	Blocks: CRAC Channel	Blocks: TPRC3 channel inhibitor and reduces NFAT nuclear translocation and cytokine production	[234]
SKF-96365	C_22_H_26_N_2_O_3_ · HCl	Blocks: receptor-mediated Ca^2+^ entry	Blocks: voltage-gated Ca^2+^ entry; TRPC channel	[235]
GSK-5498A	C_18_H_11_F_6_N_3_O	Blocks: CRAC channel	-	[223]
GSK-5503A	C_23_H_17_F_2_N_3_O_2_	Blocks: CRAC channel	-	[224]
GSK-7975A	C_18_H_12_F_5_N_3_O_2_	Blocks: Orai1	-	[222]
Synta66	C_20_H_17_FN_2_O_3_	Blocks: CRAC channel	No effect on L-type Ca^2+^ channel either TRPC1/5	[236]
Pyr6	C_17_H_9_F_7_N_4_O	Blocks: SOCE	Effect on carbachol-induced, TRPC3-mediated calcium entry inhibits the typical Orai downstream signaling cascades in RBL mast cells (NFAT activation and degranulation)	[219]
Pyr10	C_18_H_13_F_6_N_3_O_2_S	Blocks: CRAC channel	TRP cation 3 (TRPC3) inhibitor	[219]
RO2959	C_21_H_19_F_2_N_5_OS.xHCl	Blocks: SOCE	Potent inhibitor of human IL-2 production, potently blocks T-cell receptor-triggered gene expression and T-cell functional pathways	[231]

**Table 2:** Expression, localization, and biological functions of CRAC channel components and K_Ca2+_ channels individually as well as complexed either with each other or with accessory proteins. This table depicts the expression, localization, and biological functions of CRAC and K_Ca2+_ ion channels in normal and related cancer tissue. Moreover, disease-related mutations of the proteins are summarized.

Channel Type or Channel-Protein Complexes	Expression Level		Localization		Associated Mutation	Biological Function		Ref
	Normal Tissue	Cancer Tissue	Normal Tissue	Cancer Tissue		Normal Tissue	Cancer Tissue	
**Orai1 channels**	low	high (reduced in prostate cancer)	Ubiquitously expressed in a diversity of tissues Heart brain kidney lung skeletal muscle and other organs	Renal carcinoma Breast Melanoma Glioma Esophageal squamous cell carcinoma Pancreatic adenocarcinoma Prostate	A137V (colorectal tumor) M139V (stomach carcinoma) S159L (uterine carcinoma) G183D (glioblastoma) G247S (neck carcinoma)	Involved in Ca^2+^ signaling indispensable role in the immune system	Proliferation Migration Increased cell survival Tumor growth progression metastasis	[61,132,251,252,253]
**Orai1-SPCA2**	-	high	-	Breast	n.d.	Separately involved in cell Ca^2+^ signaling	Activation of Ras–ERK pathway, involved in proliferation and cell cycle Tumorigenesis Cell growth	[254,255]
**Orai2 channels**	low	high	brain and at lower levels in the spleen, lung, and small intestine	Parathyroid tumors Prostate	n.d.	Involved in Ca^2+^ signaling	Proliferation	[256,257]
**Orai3 channels**	low	high	wide tissue expression including the heart, brain, kidney, lung, skeletal muscle, and other organs	Breast Prostate Renal carcinoma Lung adenocarcinoma	n.d.	Involved in Ca^2+^ signaling	Proliferation Invasion Increased cell survival Tumor growth progression Apoptosis	[59,258,259]
**STIM1**	low	high	Ubiquitously expressed in a diversity of tissues, such as heart, skeletal muscle, and the central nervous system	Breast Lung adenocarcinoma Glioblastoma Colorectal, gastric, cervical cancer renal carcinoma hepatocellular carcinoma	A79T, E87Q, W350L, G446C/V (lung adenocarcinoma) S116N (Glioblastoma)	Involved in Ca^2+^ signaling indispensable role in the immune system	Migration Invasion Increased cell survival	[260,261,262,263,264]
**STIM2**	low	high	diverse primary lymphocytes such as T_h_, T_C_, and B-cells	Glioblastoma Prostate Melanoma Colorectal cancer	n.d.	Involved in Ca^2+^ signaling	Migration Invasion	[265,266,267]
**SK channels**	low	high	Neuronal tissues, colon, corpus cavernosum, adrenal gland, brain, prostate, bladder, liver, and heart	Breast Colon Medulloblastoma Melanoma Glioma Leukemia cells	n.d.	Synaptic function. Firing in pacemaker neurons. Control the pattern of single spike firing of dopamine neurons	Migration, proliferation tumor cell dissemination, and metastasis	[38,268,269,270]
**SK3-Orai1**	low	high	guinea pig gall bladder smooth muscle	Breast	n.d	Involved in Ca^2+^ signaling regulate muscle contraction	Migration Ca^2+^-dependent invasive process Bone metastasis Constitutive Ca^2+^ entry	[34,44]
**SK3-SigmaR1-Orai1**	-	high	-	Breast Colorectal	n.d.	Involved in Ca^2+^ signaling	Migration Ca^2+^-dependent invasive process Bone metastasis Constitutive Ca^2+^ entry	[271]
**SK3-TRPC1-Orai1**	-	high	-	Colon	n.d.	Involved in Ca^2+^ signaling	Migration activation of the Akt pathway SOCE amplification Constitutive Ca^2+^ entry	[272]
**SK3-cAMP-Orai1**	-	high	-	Breast	n.d.	Involved in Ca^2+^ signaling	Migration Constitutive Ca^2+^ entry Bone metastasis	[36].
**IK channels**	low	high	Blood, microglial endothelial and epithelial cells trachea, prostate, placenta and salivary glands. presence in excitable cells such as central neurons and cardiomyocytes	Prostate Breast Glioblastoma Endometrial, hepatocellular, and cervical carcinoma	n.d.	Immune responses of B and T cells Secretion in epithelial tissues	Tumor cell signaling including cell cycle progression Proliferation, Migration and the Epithelial-Mesenchymal Transition	[19,42,273,274,275]
**BK channels**	low	high	Skeletal muscles Nervous system, Smooth muscle cells	Prostate Breast Glioblastoma Neuroblastoma	n.d.	Regulation of calcium signaling related processes	Proliferation, migration, metastasis, apoptosis	[276,277,278]

**Table 3:** SK channel modulators. This table summarizes the most common SK channel modulators together with their empirical formula, the function of interest and some additional information.

Name	Empirical Formula	Function of Interest	Additional Information	References
**1-EBIO**	C_9_H_10_N_2_O	Activates: SK1, SK2, SK3, SK4	-	[313,318,323]
**NS309**	C_8_H_4_Cl_2_N_2_O_2_	Activates: SK1, SK3, SK4	Blocks: L-type channel	[314,316,318]
**Riluzole**	C_8_H_5_F_3_N_2_OS	Activates: SK1, SK2, SK3, SK4	Activates: TRPC5 channel	[313,324]
**CyPPA**	C_16_H_23_N_5_	Activates: SK2, SK3	Blocks: TRPM7	[314,318,325]
**SKA-111**	C_12_H_10_N_2_S	Activates: SK4	-	[326]
**SKA-121**	C_12_H_10_N_2_O	Activates: SK4	-	[326]
**ICAGEN**	C_13_H_10_N_4_S	Blocks: SK1, SK2, SK3	-	[315]
**NS8593**	C_17_H_18_ClN_3_	Blocks: SK1, SK2, SK3	Blocks: TRPM7	[315,318,327]
**BMB**	C_21_H_20_BrNO_6_	Blocks: SK3	Blocks: gamma-aminobutyric acid (GABA)-gated Cl- channels	[314]
**Apamin**	C_79_H_131_N_31_O_24_S_4_	Blocks: SK1, SK2, SK3	-	[312,328]
**TRAM34**	C_22_H_17_ClN_2_	Blocks: SK4	-	[318,329]
**4-AP**	C_5_H_6_N_2_	Blocks: SK3	Blocks: Voltage gated potassium channels	[317]
**RA-2**	C_22_H_16_F_2_O_6_	Blocks: SK1, SK2, SK3, SK4	-	[330]
**Alkyl-ether-lipids**	-	Blocks: SK3	-	[38,317]

## Abbreviations

1-EBIO	1-Ethylbenzimidazolinone
2-APB	2-aminoethoxydiphenyl borate
4-AP	*4-aminopyridine*
Å	Angstrom (unit of length equal to 10^−10^ m)
aa	Amino acid
AC8	Adenylyl cyclase 8
Akt	Known as protein kinase B or PKB
ARC	Arachidonic acid (AA)-activated channels
ATP	Adenosine triphosphate
ANSGA	4-Point mutation in hinge region aa position 261–265
ATPase	Adenosine triphosphatase
BK	Large conductance, Ca^2+^-activated potassium channels
BTP2	[*N*-{4-[3,5-bis(Trifluoromethyl)-1H-pyrazol-1-yl]phenyl}-4-methyl-1,2,3-thiadiazole-5-carboxamide]
Ca^2+^	Calcium ion
CAD	Ca^2+^ release-activated Ca^2+^-activating domain
CaM	Calmodulin
cAMP	Cyclic adenosine monophosphate
CAR	Ca^2+^ accumulating region
CaV1	Caveolin-1
CC	Coiled-coil
Ccb9	Coiled-coil domain containing region b9
cEF	Canonical EF hand
CRAC	Ca^2+^ release-activated Ca^2+^
CRACR2A	Calcium release activated channel regulator 2A
CRISPR/Cas9	Clustered regularly interspaced short palindromic repeats/Cas
Cs^+^	Cesium ion
Cyppa	*N*-Cyclohexyl-*N*-[2-(3,5-dimethyl-pyrazol-1-yl)-6-methyl-4 pyrimidinamine
∆	Represents deletion mutants
*dOrai*	*Drosophila melanogaster* Orai
DVF	Divalent-free
EDA	Ectodermal dysplasia
EGF	Epidermal growth factor
EGTA	Ethylene glycol tetraacetic acid
ER	Endoplasmic reticulum
ERα	Estrogen receptor-α
ERK1/2	Extracellular-signal-regulated kinases 1 and 2
ETON	Extended transmembrane Orai1 N-terminal
FCDI	Fast calcium-dependent inactivation
FRAP	Fluorescence recovery after photobleaching
FRET	Fluorescence resonance energy transfer
GBM	Glioblastoma multiforme cells
GoF	Gain of function
GSK	GlaxoSmithKline compounds
HEK	Human embryonic kidney
I/V	Current voltage relationship
I_Ca2+_	CRAC current
I_Na+_	Sodium current in sodium divalent free solution
ID	Inhibitory domain
IH	Inhibitory helix
IK	Intermediate Ca^2+^-activated K^+^ channels
IP3	Inositol-triphosphate
IP3R	Inositol-triphosphate receptor
K^+^	Potassium ion
K_Ca_	Ca^2+^-activated K^+^ channels
Kir	Inward-rectifier potassium channels
L1-L3	Loop 1–3 (of Orai channels)
L-type	Long-lasting calcium channel
LGC	Ligand gated calcium channels
LoF	Loss of function
MCU	Mitochondrial Ca^2+^ uniporter
MD simulations	Molecular dynamics simulations
Na^+^-DVF	Sodium divalent free
nEF	Non-canonical EF hand
NFAT	Nuclear factor of activated T cells
nAChR	Nicotinic acetylcholine receptors
NS309	3-Oxime-6,7-dichloro-1H-indole-2,3-dione
NS3893	*N*-[(1R)-1,2,3,4-Tetrahydro-1-naphthalenyl]-1H-Benzimidazol-2-amine hydrochloride
NMR	Nuclear magnetic resonance
OASF	Orai-activating small fragment
Orai 1–3	Orai proteins (also as O1–3)
P/S	Proline, serine
P-Akt	Phosphorylated known as protein kinase B or PKB
PI3K	Phosphoinositide 3-kinases
PIP_2_	Phosphatidylinositol 4,5-bisphosphate
PKA	Protein kinase A
PKC	Protein kinase C
PM	Plasma membrane
PMCA	Plasma membrane Ca^2+^ ATPase
Ref	References
S	Signal peptide
SAM	sterile α-motif
SARAF	Store-operated calcium entry associated regulatory factor
SCDI	Slow calcium-dependent inactivation
SCID	Severe combined immune deficiency
SigmaR1	Sigma non-Opioid intracellular receptor 1
SK channels	Small-conductance Ca(2+)-activated K(+) channels
SKA-111	5-Methylnaphtho[1¨C-d]thiazol-2-amine
SKA-121	5-Methylnaphth[2,1-d]oxazol-2-amine
SOAP	STIM-Orai association pocket
SOAR	*STIM*-Orai activating region
SOC	Store operated channel
SOCE	Store-operated calcium entry
SPCA2	Secretory pathway Ca^2+^-ATPase
STIM	Stromal interaction molecule
STRMK	Stormorken syndrome
Synta66	4-Pyridinecarboxamide
TAM	Tubular Aggregate Myopathy
TM	Transmembrane helices
TRP	Transient receptor potential ion channel (C-canonical, M-melastatin, V-vallinoid)
VGCC	Voltage gated calcium channels
